# Dual‐Responsive Dynamic Covalent Bond‐Based Assembly of Lipid‐Nanozyme Systems via Multi‐Target Synergy and Efficient Target Enrichment for Ischemic Stroke Therapy

**DOI:** 10.1002/advs.202519226

**Published:** 2026-04-07

**Authors:** Mengcheng Guo, Qingran Guan, Guanyu Qiao, Lixue Zhang, Man Liu, Zhen Li, Qingbiao Yang, Meili Shen, Linlin Liu, Yapeng Li

**Affiliations:** ^1^ Engineering Research Center of High Performance Plastics College of Chemistry Ministry of Education Jilin University Changchun Jilin China; ^2^ Department of Radiation Oncology China‐Japan Union Hospital of Jilin University Changchun Jilin China; ^3^ College of Chemistry Jilin University Changchun Jilin China

**Keywords:** blood brain barrier penetration, dual‐responsive nanozymes, ischemic stroke, neural repair, oxidative stress–inflammatory–apoptotic

## Abstract

To address the key challenges in ischemic stroke treatment—inadequate multi‐target intervention, low blood‐brain barrier (BBB) permeability, and lack of lesion‐specific drug release—we developed an innovative pH/ROS dual‐responsive lipid‐nanozyme system, PBB@AHA. The system features a biomimetic phospholipid dual‐tail structure that dynamically integrates the iNOS inhibitor N3, the neuroprotective agent PCA, and a vitamin E‐based derivative VES‐APBA via dynamic covalent bonding, followed by electrostatic self‐assembly with Prussian blue nanozyme (PBB) to form a synergistic therapeutic platform. Notably, VES‐APBA leverages its lipid‐like properties to efficiently cross the BBB through passive diffusion and active transport, achieving effective accumulation at the lesion site. Under the characteristic acidic pH and elevated ROS levels of the ischemic microenvironment, the dual‐responsive bonds cleave, triggering synchronous release of PBB (for ROS scavenging), N3 (for anti‐inflammation), and PCA (for anti‐apoptosis and neuroprotection), thereby collectively blocking the oxidative stress–inflammation–apoptosis multi‐target pathway. In a rat model, PBB@AHA significantly reduced infarct volume by 81.7 ± 3.3% and improved neurological function scores by 73.2 ± 4.1%, offering a novel and precise nanomedicine strategy for stroke treatment.

## Graphical abstract

This system adopts a biomimetic phospholipid structure, covalently binding iNOS inhibitors, neuroprotective agent PCA and vitamin E derivatives through pH/ROS dual‐responsive bonds, and assembles them with Prussian blue nanozyme to form PBB@AHA. It can efficiently penetrate the blood‐brain barrier and simultaneously release multiple active components in response to the pathological microenvironment in the lesion area, achieving synergistic multi‐target therapy.

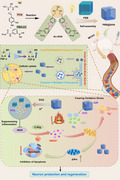

## 1 Introduction

Ischemic stroke (IS), a high‐risk critical condition in the field of neurology, initiates a pathological cascade when local ischemia and hypoxia occur due to hemodynamic disturbances in cerebral arteries [1]. When cerebral blood vessels are blocked, depriving brain cells of oxygen and glucose (OGD), mitochondrial oxidative phosphorylation is hindered, ATP synthesis fails, and energy metabolism is disrupted [2]. This metabolic imbalance triggers a vicious cycle through dual pathways: on one hand, reactive oxygen species (ROS) accumulate explosively via the nicotinamide adenine dinucleotide phosphate (NADPH) oxidase and xanthine oxidase pathways, driving oxidative stress; on the other hand, enhanced anaerobic glycolysis leads to intracellular acidosis (pH < 6.5) and the release of numerous inflammatory factors (such as TNF‐α, IL‐1β), strongly activating inducible nitric oxide synthase (iNOS), resulting in excessive nitric oxide (NO) production [3, 4, 5]. The peroxynitrite (ONOO^−^) formed by the reaction of NO with ROS intensifies neuroinflammation, causes mitochondrial membrane potential collapse, and activates the mitochondrial‐dependent apoptotic pathway, ultimately leading to irreversible neuronal damage and programmed cell death [6, 7].

In the pathological cascade of ischemic stroke, the interaction among the oxidative stress–inflammation–apoptosis axis is complex. Traditional therapeutic approaches only target a single link in this pathological axis, resulting in limited efficacy and an inability to effectively interrupt the vicious cycle of the cascade reaction. For instance, traditional Prussian blue nanozyme (PBB) can efficiently simulate the functions of catalase (CAT) and superoxide dismutase (SOD) to clear ROS and maintain redox homeostasis, but it is ineffective in suppressing the excessive generation of NO mediated by iNOS and the subsequent toxicity of ONOO^−^ and neuroinflammation [8, 9]. Similarly, 1,3‐diaminoguanidine (N3), as a potent iNOS inhibitor, can block iNOS activity, significantly reduce NO levels, inhibit ONOO^−^ formation and neuroinflammation, but it has insufficient ability to clear accumulated ROS and limited regulatory effect on the downstream apoptotic pathway [10]. Protocatechuic aldehyde (PCA) has been proven to have neuroprotective effects (improving microenvironment, clearing ROS, anti‐inflammation, and anti‐apoptosis), but its single‐target therapy is still insufficient in dealing with the complex multi‐pathway damage [11, 12, 13, 14, 15, 16].

The development of multi‐target nanotherapeutic systems capable of simultaneously targeting oxidative stress, inflammation and apoptosis is an inevitable direction for improving therapeutic efficacy. However, how to break through the blood‐brain barrier (BBB) to achieve an effective therapeutic concentration of nanotherapeutic systems in ischemic brain regions remains an urgent problem to be solved. The lipid bilayer structure of BBB endothelial cells determines that highly lipophilic compounds are more easily permeable and can quickly reach a distribution equilibrium between plasma and brain tissue. However, most molecules with therapeutic potential (such as N3, PCA) have insufficient lipophilicity, which seriously hinders their delivery efficiency to the brain [17, 18, 19]. In view of this biological characteristic of the BBB, natural antioxidant lipophilic small molecules (such as vitamin E, Omega‐3 fatty acids, lipoic acid) provide important ideas for solving the delivery problem: the structural basis for their transport across the BBB has been confirmed. By modifying these molecules on the surface of nanodelivery systems, they can take advantage of the compatibility with the lipid bilayer of BBB endothelial cells and efficiently penetrate the BBB through passive diffusion and active transport to exert therapeutic effects [19, 20]. In contrast, traditional liposomes only rely on inefficient passive diffusion and are limited by particle size and the clearance effect of the reticuloendothelial system. They have single functions and can only serve as drug carriers, showing deficiencies in penetration efficiency, targeting, and functional integration [21, 22, 23]. Moreover, current research mostly focuses on improving single delivery efficiency and is difficult to achieve multi‐target synergistic intervention of the above pathological cascades. Even if the nanosystem successfully penetrates the BBB, its therapeutic efficacy will be limited due to the inability to block the vicious cycle of the pathological axis. Therefore, it is necessary to design a dual‐responsive nanotherapeutic system with both multi‐target synergy and efficient enrichment.

Herein, this study designed a pH/ROS dual‐responsive nanosystem for synergistic therapy based on dynamic covalent bonds. Inspired by the phospholipid double‐tail structure, the positively charged iNOS inhibitor N3 was successively conjugated to PCA and phenylboronic acid‐functionalized lipid derivatives (PBA‐LD) via pH‐sensitive imine bonds and ROS‐sensitive borate ester bonds, respectively, yielding phospholipid‐like liposomes (An‐AHA) with N3 as the hydrophilic head and PBA‐LD as the hydrophobic tail chain. Subsequently, N3 within An‐AHA further underwent electrostatic self‐assembly with negatively charged PBB, resulting in a series of drug‐loaded and structurally stable pH/ROS dual‐responsive nanosystems for synergistic therapy (PBB@An‐AHA). Regarding the interaction of the oxidative stress–inflammation–apoptosis axis within the pathological cascade, PBB@An‐AHA can achieve multi‐target coverage through the synergy of three components. Specifically, PBB mimics the dual enzymatic activities of CAT and SOD to scavenge ROS, thereby blocking the initiation of oxidative stress. N3 inhibits the activity of iNOS and downregulates the secretion of pro‐inflammatory factors such as TNF‐α/IL‐1β, thus curbing the spread of inflammation. PCA repairs the mitochondrial membrane potential, inhibits the apoptotic pathway, and induces the M2 polarization of microglia, thereby promoting neural repair, thereby overcoming the limitations of single‐target therapies. To address the challenge of insufficient BBB penetration, the lipophilic modification of the PBA‐LD hydrophobic tail chain (such as vitamin E) was exploited to match the lipophilic characteristics of the lipid bilayer of BBB endothelial cells. This significantly enhances the trans‐barrier efficiency of nanoparticles, ensuring the effective delivery of drugs to the lesion site. Simultaneously, leveraging the pH/ROS dual‐responsive dynamic covalent bonds, the precise and coordinated release of the three components within the ischemic microenvironment is achieved, further enhancing the therapeutic efficacy. In conclusion, this system offers a targeted and innovative solution for overcoming the treatment bottleneck of ischemic stroke through an integrated strategy encompassing “structural biomimetic design—multi‐target synergistic intervention—enhanced barrier crossing—microenvironment‐responsive release”. Moreover, it provides novel insights into the treatment of complex multi‐target diseases.

## 2 Results

### 2.1 Synthesis and Characterization of PBB@AHA

**SCHEME 1:**
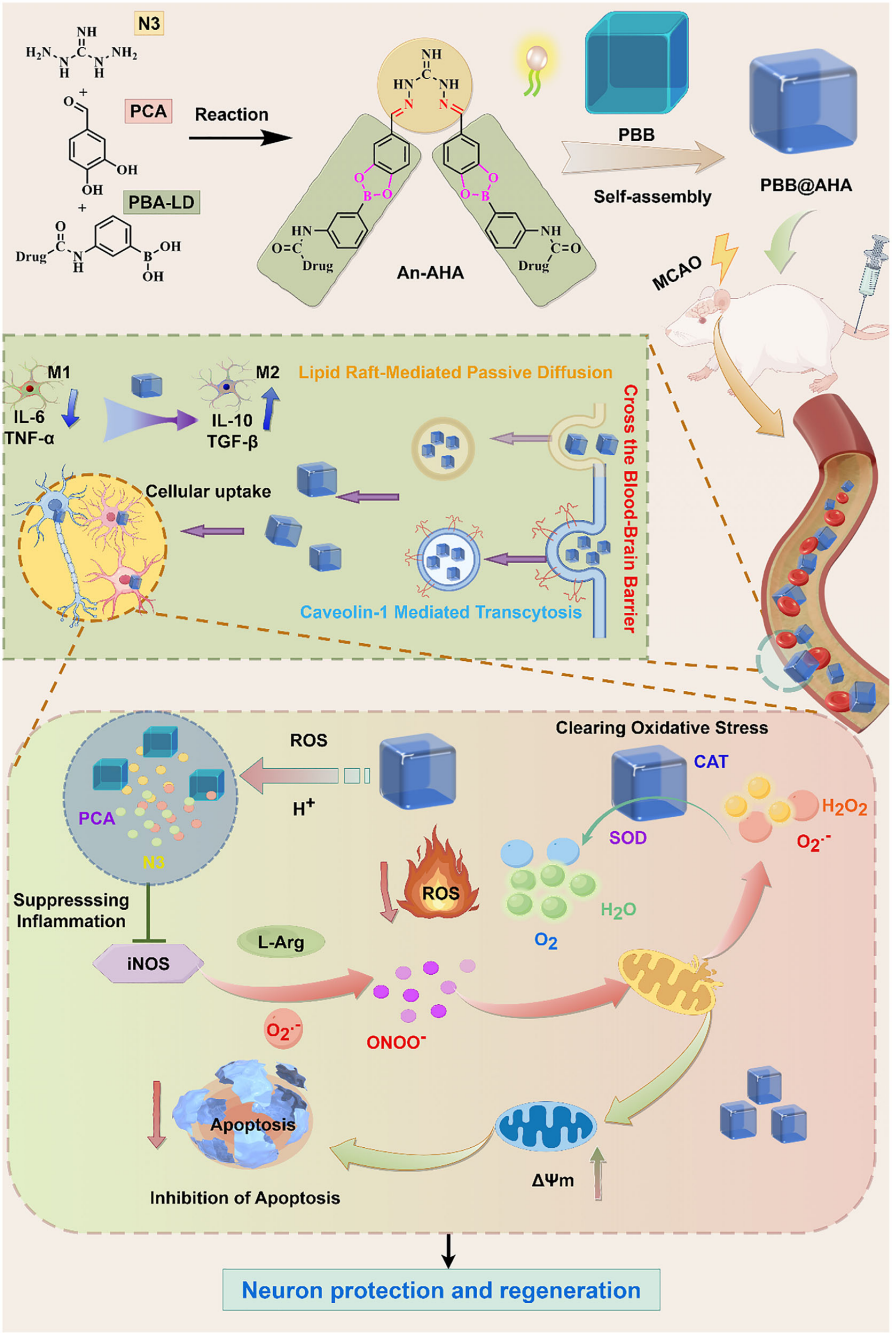
PBB@AHA Synthesis Route and Its Therapeutic Mechanism Diagram, Image Drawn by Figdraw.

**FIGURE 1:**
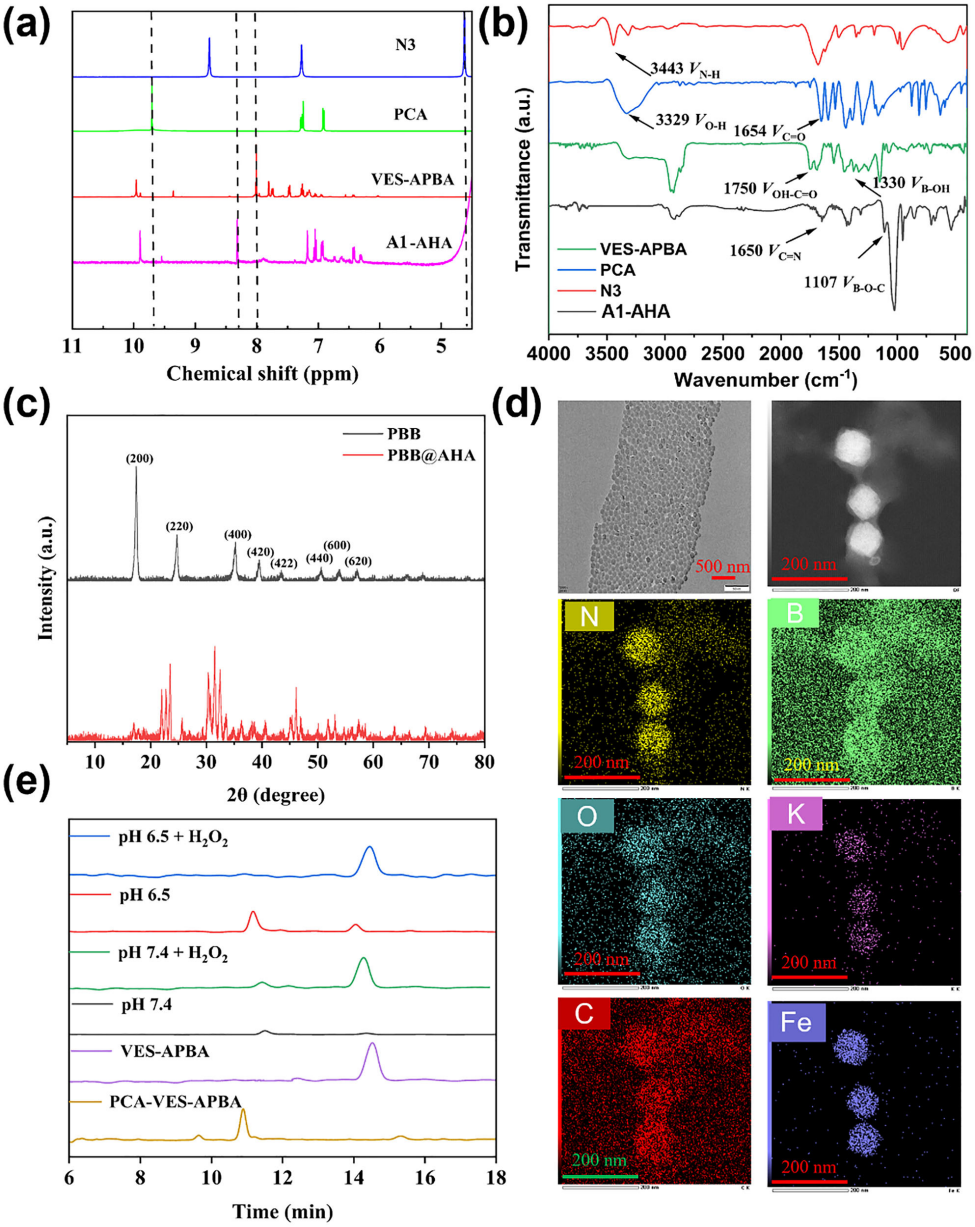
Synthesis and characterization of PBB@AHA. (a) ^1^H NMR spectra of N3, PCA, VES‐APBA, and A1‐AHA in D_2_O. (b) FTIR spectra of N3, PCA, VES‐APBA, and A1‐AHA. (c) XRD patterns of PBB and PBB@AHA. (d) Representative TEM images and corresponding elemental mapping of PBB@AHA assemblies. (e) HPLC chromatograms of PBB@AHA after 2 h exposure to PBS (pH 7.4 or 6.5) with or without H_2_O_2_ (100 µm).

In this study, a series of phenylboronic acid‐functionalized lipid derivatives (PBA‐LD, Scheme 1) were first synthesized, and their structures were verified using proton nuclear magnetic resonance spectroscopy (^1^H NMR) (Figures S1–S6). N3, PCA, and PBA‐LD were reacted in dimethyl sulfoxide (DMSO) at a molar ratio of 1:2:2, leading to the formation of phospholipid bitailed liposomes An‐AHA (*n* = 1, 2, 3, or 4) through self‐assembly. As shown in Figure S7, high‐performance liquid chromatography‐mass spectrometry (LC‐MS) confirmed the successful synthesis of An‐AHA. Furthermore, dynamic light scattering (DLS) and Zeta potential analyses revealed that the dynamic liposomes (A1‐AHA), constructed from phenylboronylated vitamin E derivatives (VES‐APBA), exhibited an optimal hydrodynamic diameter of 32.1 ± 0.4 nm, a narrow size distribution with a polydispersity index (PDI) of 0.167 ± 0.003, and a strongly electronegative surface charge (‐43.5 ± 3.6 mV). Therefore, A1‐AHA (hereafter referred to as AHA) was selected as the target system for further characterization. To confirm the chemical structure of AHA, we systematically characterized the reaction products using multidimensional spectroscopic techniques. The ^1^H NMR analysis showed that the characteristic peak of N3 (*δ* = 4.6 ppm), the aldehyde peak of PCA (*δ* = 9.7 ppm), and the characteristic group peak of VES‐APBA (*δ* = 8.0 ppm) completely disappeared from the AHA spectrum after the reaction, while a new proton signal corresponding to the imine bond appeared at *δ* = 8.3 ppm, indicating the successful formation of imine bond linkage between N3 and PCA. The presence of borate bonds in AHA was further confirmed by ^11^B NMR spectroscopy (Figure S8a): the characteristic signal at *δ* = 28.1 ppm assigned to the boric acid group (B─OH) in AHA disappeared and was replaced by a new peak corresponding to the borate bond (PhB─C) at *δ* = 8.4 ppm. Fourier transform infrared spectroscopy (FTIR) also supported the formation of dynamic covalent bonds in AHA (Figure 1b): the characteristic absorption bands at 3443 cm^−1^ (N─H stretch of N3), 3329 cm^−1^ (O─H stretch of PCA), and 1330 cm^−1^ (B─OH stretch of VES‐APBA) were no longer present in the AHA spectrum. Instead, new absorption bands emerged at 1650 cm^−1^ (C═N stretching vibration of the imine bond) and 1107 cm^−1^ (B─O─C bending vibration of the borate ester bond). Additionally, the characteristic absorption peaks of the VES‐APBA structural unit were observed at 288 nm and 331 nm in the ultraviolet–visible (UV–vis) spectrum (Figure S8b), further confirming its successful integration into the system.

Subsequently, PBB was synthesized following the PBB synthesis protocol described in the literature [21]. To significantly improve the dispersion of nanozymes, an electrostatically driven self‐assembly strategy was employed by dropwise addition of a DMSO solution containing the AHA prodrug into a phosphate buffer solution containing PBB. DLS results (Figure S8c,d) demonstrated that the hydrodynamic diameter of native PBB was 134.4 ± 0.7 nm, which decreased to 124.8 ± 1.5 nm after coating with AHA to form PBB@AHA. This reduction may be attributed to enhanced dispersion during the coating process. Concurrently, the Zeta potential of PBB@AHA shifted from ‐13.2 ± 0.5 mV for uncoated PBB to ‐38.7 ± 2.1 mV, confirming that the AHA coating substantially increased the surface negative charge. X‐ray diffraction (XRD) analysis revealed that PBB@AHA retained all the characteristic diffraction peaks corresponding to the cubic crystal structure of PBB (Figure 1c), indicating that the composite formation did not compromise the crystallinity of the nanozyme. Transmission electron microscopy (TEM) imaging showed that PBB@AHA maintained well‐defined cubic morphology, with particle size consistent with DLS measurements. Elemental mapping further confirmed uniform distribution of Fe, B, C, N, O, and K within the assemblies (Figure 1d). Notably, PBB@AHA exhibited excellent colloidal stability, as evidenced by minimal changes in particle size after storage in PBS at 4°C for 35 d (Figure S8e), thereby establishing a solid foundation for subsequent experimental investigations.

In response to the pathological features of the ischemic reperfusion injury lesion area, such as high‐concentration lactic acid accumulation (leading to local acidification) and excessive ROS expression, we systematically evaluated the microenvironment‐responsive drug release behavior of the PBB@AHA nano‐assembly and further explored the release mechanism of VES‐APBA within the AHA assembly. The results showed (Figure S8f) that due to the H_2_O_2_‐responsive property of the borate ester bond, H_2_O_2_ could significantly trigger the release of VES‐APBA. While a single acidic environment (pH 6.5) could also promote the release of VES‐APBA to a certain extent, but the amount was limited. This might be due to the generation of free PCA‐VES‐APBA containing the VES‐APBA structure. Notably, when pH 6.5 and H_2_O_2_ acted together, the release rate of VES‐APBA increased significantly, and the release amount of VES‐APBA exceeded 70% at 16 h, confirming its specific response to the complex pathological microenvironment of the lesion. Similar results were also observed in subsequent Zeta potential and hydrodynamic diameter characterizations (Figure S8c,d). PBB@AHA presented a stable colloidal state under physiological conditions (pH 7.4), with a Zeta potential of ‐38.7 ± 2.1 mV and a particle size of 124.8 ± 1.5 nm. However, an acidic environment (pH 6.5) could induce a further negative shift in its Zeta potential to ‐45.8 ± 3.7 mV, and the particle size increased to 187.3 ± 4.3 nm, indicating partial structural dissociation of the assembly. Treatment with H_2_O_2_ also triggered dissociation, increasing the particle size to 162.3 ± 5.4 nm under pH 7.4 conditions. These structural dissociation phenomena created necessary conditions for drug release. Based on the above structural change characteristics, we further utilized high‐performance liquid chromatography (HPLC) to measure the release of VES‐APBA (Figure 1e). After exposing PBB@AHA to pH 6.5/H_2_O_2_ or H_2_O_2_, an elution peak of VES‐APBA appeared at 14 min, confirming that H_2_O_2_ drives the release of VES‐APBA by inducing the cleavage of the borate ester bond. While the single acidic condition mainly triggers the release of free PCA‐VES‐APBA containing the VES‐APBA structure. In conclusion, our PBB@AHA component can release VES‐APBA under H_2_O_2_ and acidic conditions, enhancing the specific on‐demand release of drugs in the lesion microenvironment.

### 2.2 The Antioxidant Capacity of PBB@AHA

**FIGURE 2:**
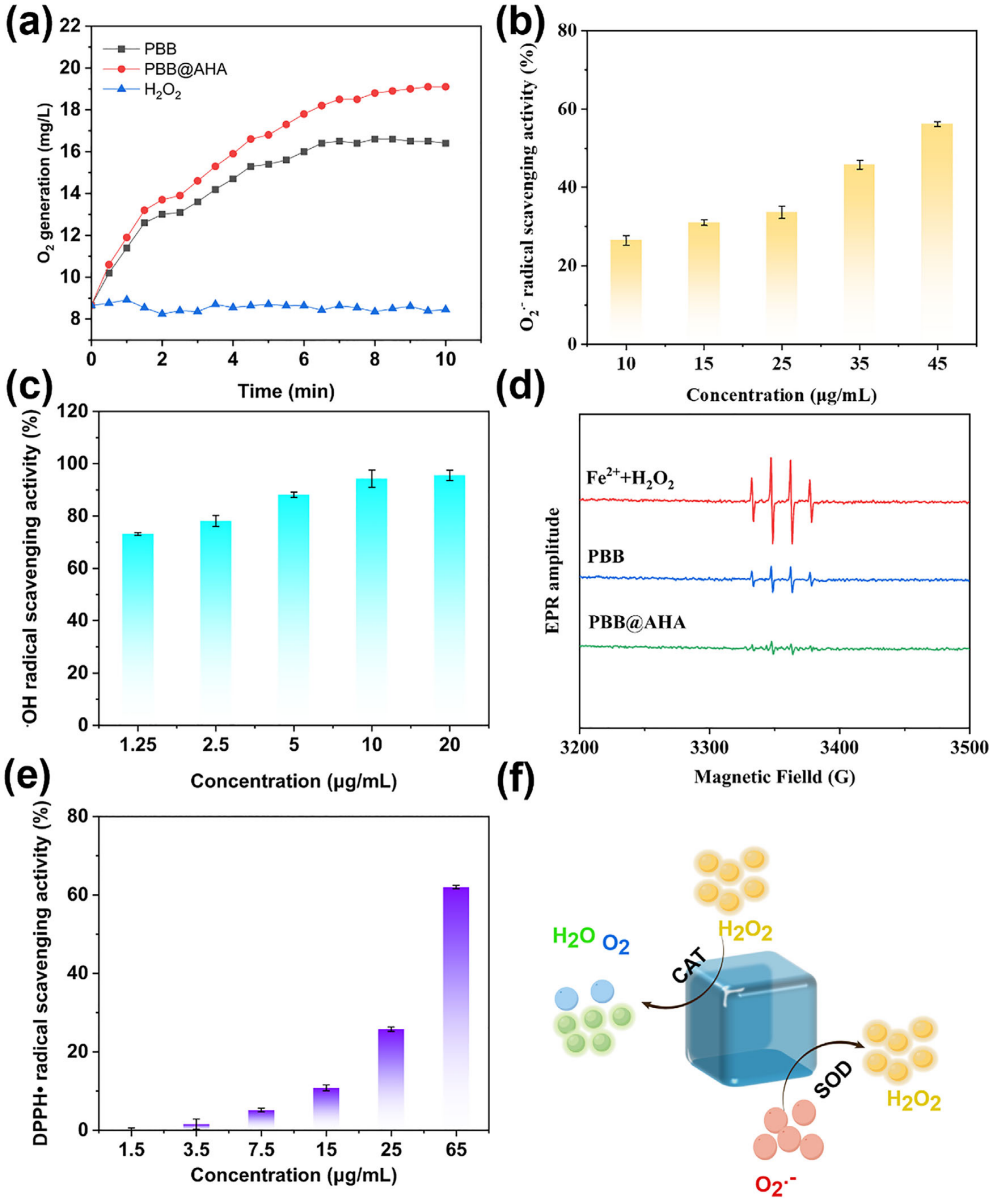
In vitro antioxidant capacity of PBB@AHA. (a) The ability of PBB@AHA to catalyze O_2_ generation from H_2_O_2_ within 10 min was measured using an oxygen meter. (b) Scavenging activity of varying concentrations of PBB@AHA against O_2_^•−^ (*n* = 3). (c) Scavenging activity of varying concentrations of PBB@AHA against •OH (*n* = 3). (d) EPR spectra employing DMPO as a spin trap to evaluate the •OH scavenging capacity across different treatment groups. (e) Scavenging activity of varying concentrations of PBB@AHA against DPPH• (*n* = 3). (f) Schematic illustration of the antioxidant mechanism of PBB@AHA.

To systematically evaluate the enzymatic properties and antioxidant capacity of PBB@AHA, this study employed multidimensional spectroscopic methods for characterization. To confirm the CAT‐like activity of PBB@AHA, its ability to catalyze the decomposition of H_2_O_2_ into O_2_ was monitored using an oxygen meter, which directly reflects the CAT‐like function. The results showed (Figure 2a) that PBB@AHA efficiently catalyzed the decomposition of H_2_O_2_ to produce O_2_, with catalytic efficiency comparable to that of PBB, indicating that the nano‐system retained the intrinsic CAT‐like activity of PBB after AHA loading. To verify the SOD‐like activity of the PBB@AHA nanozyme, a superoxide anion (O_2_^•−^) scavenging assay was conducted. Results demonstrated that 45 µg mL^−1^ of PBB@AHA could eliminate approximately 53% of O_2_^•−^ (Figure 2b), confirming its notable SOD‐like activity. Additionally, the hydroxyl radical (•OH) scavenging efficiency was quantitatively assessed using the salicylic acid‐ultraviolet‐visible spectrophotometry method. In the Fenton reaction (FeSO_4_/ H_2_O_2_), the generated •OH reacts with salicylic acid to form 2,3‐dihydroxybenzoic acid, which exhibits a characteristic absorption peak at 510 nm. As shown in Figure 2c, upon adding as little as 1.25 µg mL^−1^ of PBB@AHA, the •OH scavenging efficiency reached up to 73.2 ± 2.4%. Electron paramagnetic resonance (EPR) analysis (Figure 2d) further confirmed ROS scavenging capability. Upon addition of either PBB or PBB@AHA to the Fe^2+^/H_2_O_2_ system, the characteristic signal intensity of •OH was reduced in both cases. However, compared with equimolar PBB, PBB@AHA induced a more pronounced decrease in signal intensity, suggesting that AHA loading enhances ROS scavenging to some extent. Subsequently, 1,1‐diphenyl‐2‐picrylhydrazyl (DPPH•) was used to assess total ROS scavenging capacity. After incubation with PBB@AHA, the characteristic DPPH• signal was significantly attenuated (Figure 2e), and the scavenging efficiency exhibited a dose‐dependent trend. These experimental findings demonstrate that PBB@AHA possesses multiple enzyme‐like catalytic activities, including superoxide dismutase SOD‐like and catalase CAT‐like functions. Moreover, they highlight the considerable potential of PBB@AHA as an efficient and broad‐spectrum antioxidant. Therefore, it offers a promising nanozyme‐based therapeutic strategy for mitigating oxidative stress during ischemia‐reperfusion injury.

### 2.3 PBB@AHA Enhances BBB Penetration and Targeted Enrichment in Cerebral Ischemic Lesions

**FIGURE 3:**
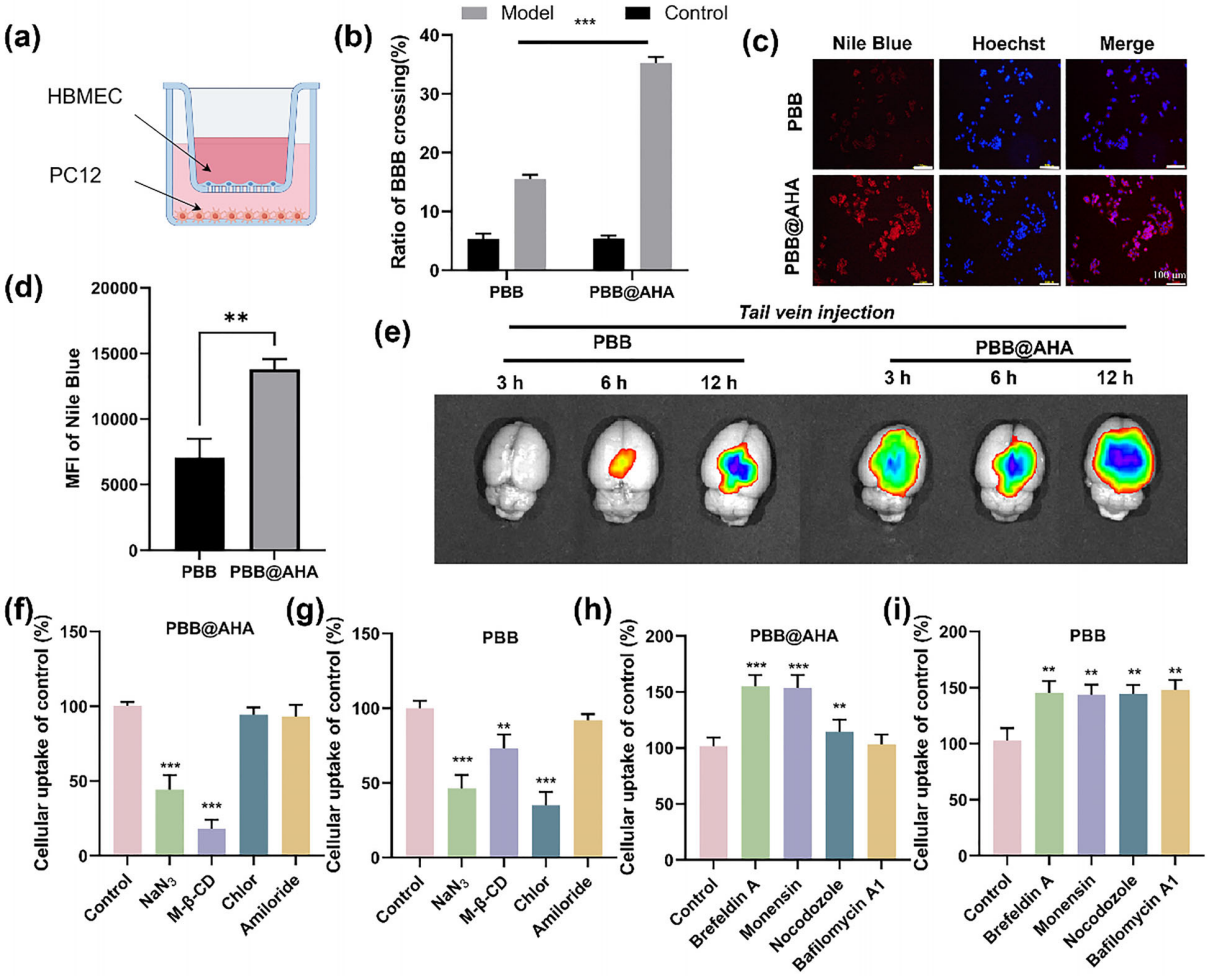
The ability of PBB@AHA to penetrate the BBB. (a) Schematic illustration of the Transwell model setup for simulating BBB penetration by PBB@AHA (upper chamber: HBMEC cells; lower chamber: PC12 cells). (b) BBB penetration rates of PBB and PBB@AHA after 12 h (*n* = 3). Inverted fluorescence microscope images of PC12 cells in the lower chamber of transwell taking up PBB and PBB@AHA(c) and their fluorescence quantification graphs (d, *n* = 3). (e) Representative ex vivo IVIS images of brains from MCAO rat models after intravenous administration of PBB or PBB@AHA. (f, g) Inhibition of cellular uptake by various endocytosis inhibitors. (h, i) Suppression of exocytosis mediated by distinct exocytosis inhibitors.

In the pathological microenvironment of ischemic stroke, although oxidative stress‐induced disruption of the BBB can transiently increase macromolecular permeability, the accompanying inflammatory cytokine storm exacerbates barrier dysfunction through a cascade reaction. This dynamic pathological feature presents a dual challenge to the targeting efficiency of nanodelivery systems: they must not only penetrate the BBB but also achieve high enrichment at the lesion site. To address this challenge, we constructed a surface‐functionalized PBB@AHA nanozyme in this study. To verify its ability to cross the BBB, an evaluation was conducted using the transwell model. As shown in Figure 3a, under physiological conditions, there was no significant difference in BBB penetration rates between PBB and PBB@AHA. In the H_2_O_2_‐induced oxidative stress environment, PBB@AHA exhibited a BBB penetration rate of 35.2 ± 3.2%, which was 2.3‐fold higher than that of PBB (Figure 3b). Furthermore, neuronal uptake efficiency of PBB@AHA was 3.2 times greater than that of PBB (Figure 3c,d), demonstrating that AHA modification enhances the ability of nanocarriers to penetrate the BBB and facilitates intracellular internalization. To further analyze the in vivo distribution pattern of the nanocarrier, we dynamically tracked the spatiotemporal distribution of nanoparticles in the brain using an in vivo imaging system (IVIS). The results in Figure 3e and Figure S10 showed that 3 h after intravenous injection, the fluorescence signal intensity of PBB@AHA in the ischemic cerebral hemisphere was 4.7 times higher than that in the PBB group, demonstrating that the pathological microenvironment‐triggered passive penetration significantly enhances the trans‐BBB transport efficiency of PBB@AHA. Notably, 6 h after injection, the fluorescence intensity ratio of the ischemic hemisphere to the contralateral healthy hemisphere reached 5.8:1, highlighting the lesion‐enriching characteristics of PBB@AHA. Further observation revealed that the fluorescence intensity on the ischemic side remained stable for up to 12 h, which was significantly better than that observed in the PBB group, indicating that AHA modification prolongs the retention time of PBB at the lesion site. Collectively, these findings indicate that PBB@AHA enhances delivery efficiency to brain lesions through a dual‐strategy approach. On one hand, it exploits the passive penetration capability in the damaged BBB region to achieve initial lesion accumulation. On the other hand, AHA modification promotes cellular internalization and retention, thereby extending the local action duration. This synergistic mechanism provides valuable insights for the development of novel nanomaterials for the treatment of ischemic brain injury.

To thoroughly investigate the underlying mechanisms contributing to the superior performance of PBB@AHA, this study employed pharmacological inhibitors to systematically compare the endocytic pathways, intracellular transport, and exocytosis processes between PBB@AHA and conventional PBB. This approach aimed to elucidate the synergistic mechanism of passive diffusion and active transport. At the level of endocytic mechanisms (Figure 3f,g), NaN_3_ (an energy depletion agent) significantly reduced the cellular uptake of both PBB and PBB@AHA, indicating that both processes are energy‐dependent and consistent with the characteristics of active transport [22]. Methyl‐β‐cyclodextrin (M‐β‐CD, a lipid raft disruptor) exhibited a significantly stronger inhibitory effect on PBB@AHA compared to PBB. In contrast, chlorpromazine (Chlor, a clathrin inhibitor) markedly suppressed PBB uptake without affecting PBB@AHA, suggesting that PBB@AHA primarily utilizes the lipid raft pathway. Lipid rafts, as lipid‐enriched membrane microdomains, serve as key mediators of passive diffusion, whereas PBB relies on clathrin‐mediated endocytosis. Amiloride (Amiloride, a macropinocytosis inhibitor) had minimal impact on both formulations, thereby excluding the involvement of macropinocytosis [23]. Collectively, these findings indicate that AHA modification enables PBB@AHA to penetrate via the “lipid raft–Caveolin‐1 specific pathway,” which is highly compatible with the lipid characteristics of VES‐APBA. This pathway enhances passive diffusion through lipid raft mediation while maintaining the energy dependence of active transport, thereby distinguishing it from the “clathrin‐dependent energy endocytosis” mechanism of PBB.

Building upon the observed differences in endocytic pathways, this study further employed exocytosis inhibitors to analyze the intracellular transport and exocytosis dynamics of both systems (Figure 3h,i). Brefeldin A (an inhibitor of ER‐to‐Golgi transport) and Monensin (an inhibitor of Golgi‐to‐plasma membrane transport) both reduced the exocytosis of PBB and PBB@AHA, indicating the involvement of the endoplasmic reticulum and Golgi apparatus in the transport process. Notably, Nocodazole (a lysosomal transport inhibitor) and Bafilomycin A1 (a lysosomal acidification inhibitor) significantly suppressed the exocytosis of PBB but not PBB@AHA, suggesting that PBB relies on lysosomal function, whereas PBB@AHA does not. Taken together, AHA modification not only reshapes the endocytic pathway but also optimizes intracellular trafficking [24]. This enables PBB@AHA to retain the energy advantage of active transport while enhancing penetration efficiency through lipid raft‐mediated passive diffusion, establishing an “active–passive cooperative transport” model. This mechanism provides a critical foundation for the efficient penetration of PBB@AHA across the BBB based on the lipid compatibility of VES‐APBA, offering valuable theoretical insights for the design and development of advanced nanomedicines.

### 2.4 PBB@AHA Inhibits Cell Apoptosis In Vitro

**FIGURE 4:**
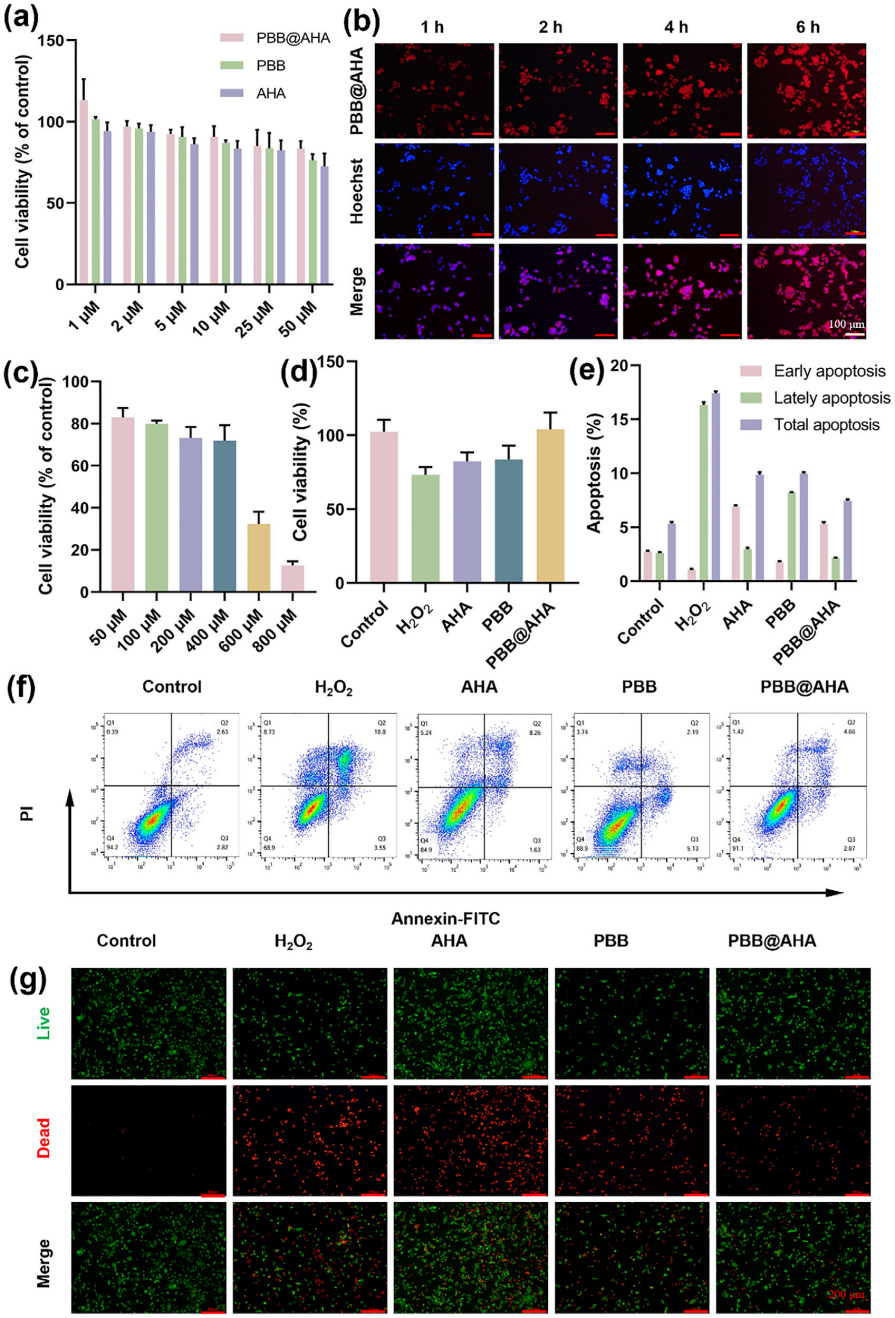
PBB@AHA protects cells from oxidative stress damage. (a) Cytotoxicity of different concentrations of AHA, PBB, and PBB@AHA after co‐incubation with PC12 cells for 24 h detected by MTT assay (*n* = 5). (b) Uptake of Nile red‐labeled PBB@AHA (red, 20 µg mL^−1^) by cells (scale bar = 100 µm). (c) Survival rate of PC12 cells after co‐incubation with different concentrations of H_2_O_2_ (*n* = 5). (d) Survival rate of PC12 cells after co‐treatment with 600 µm H_2_O_2_, AHA, PBB, and PBB@AHA (*n* = 5). The reversal effect of AHA, PBB, and PBB@AHA on H_2_O_2_‐induced cell apoptosis was evaluated using Annexin V‐FITC/PI: (e) Quantitative statistical chart of apoptosis; (f) Flow cytometry scatter plot (*n* = 3). (g) Fluorescence microscopic images of double‐stained PC12 cells with calcein AM/PI after a 24 h treatment with AHA, PBB, or PBB@AHA induced by H_2_O_2_ (scale bar = 200 µm).

In this study, PC12 cells with neuroendocrine features, derived from rat adrenal pheochromocytoma, were selected to establish an oxidative stress injury model for systematically evaluating the biosafety and antioxidant capacity of PBB@AHA. The MTT assay demonstrated that within the concentration range of 0–200 µg mL^−1^, AHA, PBB, and PBB@AHA did not exhibit significant cytotoxicity after 24 h of co‐culture with PC12 cells (Figure 4a), confirming the good biocompatibility of the material system. Fluorescence microscopic imaging revealed that Nile red‐labeled PBB@AHA was efficiently internalized by PC12 cells in a time and concentration‐dependent manner (Figure 4b and Figure S11), indicating its potential for intracellular delivery and functional activity. In the oxidative injury model induced by 600 µM H_2_O_2_, the survival rate of PC12 cells decreased to 32.1 ± 1.3% (Figure 4c). Following treatment with AHA, PBB, or PBB@AHA, cell viability was significantly restored, with PBB@AHA showing the most pronounced protective effect (Figure 4d). Given that apoptosis is a primary mechanism through which hydrogen peroxide inhibits cell growth, Annexin V‐FITC/PI staining combined with flow cytometry was employed to assess the anti‐apoptotic effects of different treatments. Results showed a marked increase in late apoptotic cells following H_2_O_2_ exposure, whereas the apoptosis rate in the PBB@AHA treatment group decreased to 6.8 ± 0.9%, significantly lower than that observed in the single‐component treatment groups (Figure 4e,f). Additionally, live/dead staining using Calcein AM and PI confirmed the protective effect of PBB@AHA through fluorescence imaging analysis (Figure 4g). These findings collectively indicate that AHA modification significantly enhances the cellular uptake efficiency of PBB, and the synergistic interaction between AHA and PBB markedly improves the antioxidant and anti‐apoptotic capabilities of PBB@AHA within PC12 cells.

Cell apoptosis is closely associated with mitochondrial dysfunction. As the central organelle of cellular energy metabolism, mitochondria produce more than 80% of ATP through oxidative phosphorylation. Moreover, as a key regulator of cellular signaling, mitochondria also serve as the primary site for the generation of reactive ROS [25, 26]. Under pathological conditions, excessive accumulation of ROS can induce mitochondrial oxidative stress, resulting in a significant reduction in mitochondrial membrane potential (ΔΨm) [27]. This, in turn, activates the Caspase cascade and ultimately initiates programmed cell death. Therefore, ΔΨm is not only a dynamic marker for assessing mitochondrial functional integrity but also serves as a molecular link between oxidative stress and apoptosis execution. Changes in its levels directly reflect the critical transition point of cell fate from metabolic imbalance to apoptosis [28]. To evaluate the effects of different treatment groups on mitochondrial function, we employed the JC‐1 fluorescent probe to monitor alterations in mitochondrial membrane potential. Red fluorescence indicates healthy mitochondria, whereas green fluorescence reflects a reduction in ΔΨm and mitochondrial damage. By measuring the ratio of average red to green fluorescence intensities, changes in mitochondrial ΔΨm can be quantitatively assessed. The experimental results showed (Figure S12) that H_2_O_2_ stimulation caused a significant decrease in ΔΨm, as evidenced by a marked increase in green fluorescence. Although treatment with AHA or PBB alone partially restored ΔΨm (to 20.3 ± 2.4% and 48.1 ± 1.9% of the control group, respectively), the PBB@AHA treatment significantly enhanced the recovery rate to 87.1 ± 0.7%, nearly reaching normal levels. These findings further confirm that PBB@AHA efficiently scavenges excessive ROS in mitochondria through its synergistic SOD/CAT‐like dual‐enzyme activity, interrupts the oxidative stress cascade, and thereby exerts a potent neuroprotective effect.

### 2.5 PBB@AHA Inhibits the Oxidative Stress–Inflammation Axis In Vitro

**FIGURE 5:**
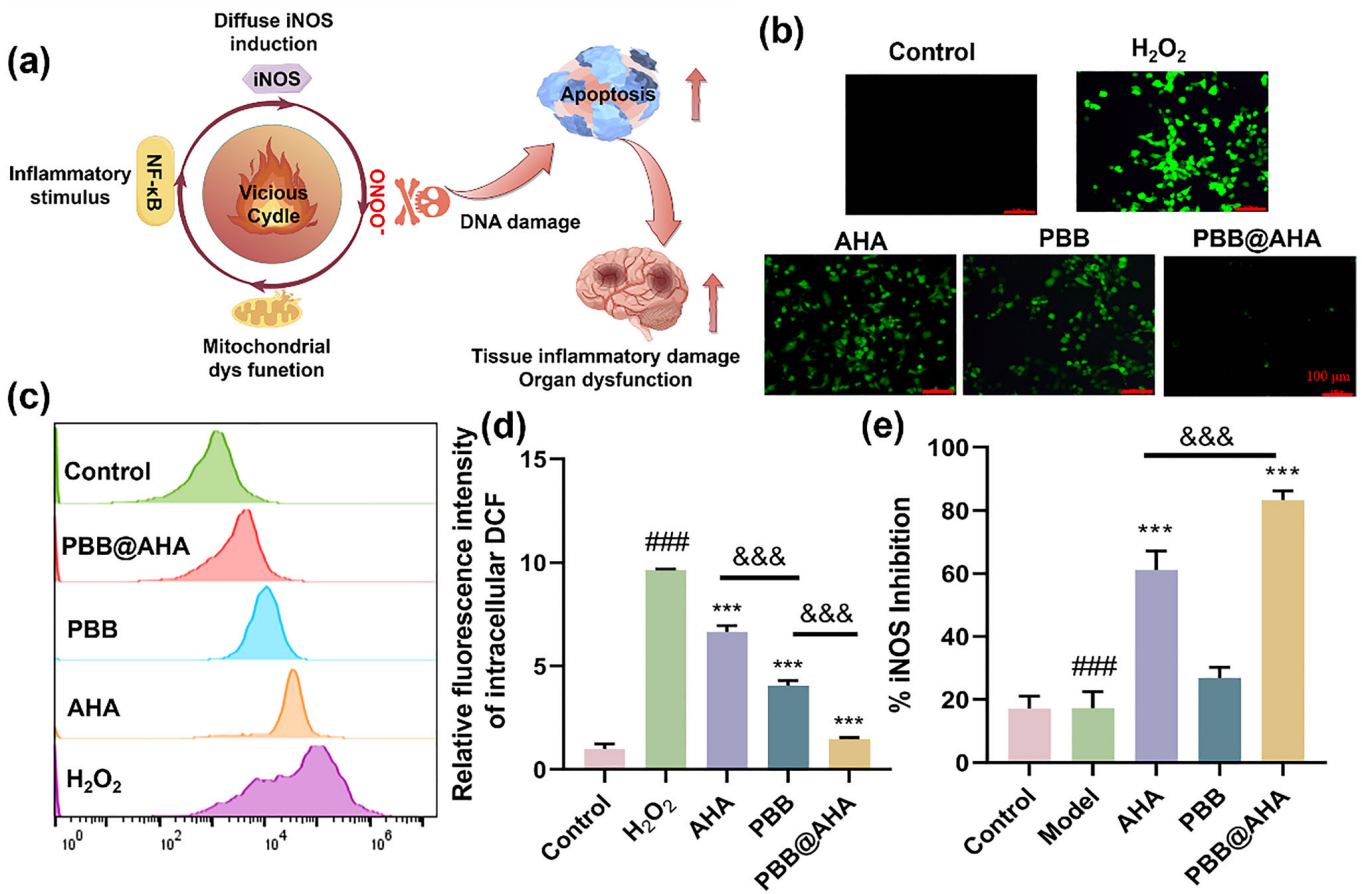
PBB@AHA inhibits the oxidative stress–inflammation axis in vitro. (a) Schematic diagram illustrating the vicious cycle mechanism of oxidative stress–inflammation. (b) Representative images of intracellular ROS levels detected using the DCFH‐DA fluorescent probe (scale bar = 100 µm). (c) Flow cytometry was used to detect intracellular ROS levels. (d) After co‐culturing PC12 cells with different drugs for 24 h, DCFH‐DA staining was performed, and DCF was quantified via flow cytometry (*n* = 3). (e) Inhibition rate of iNOS in the RAW 264.7 cell model induced by LPS/IFN‐γ after co‐incubation with AHA, PBB, and PBB@AHA, respectively. Significant differences between the control group and the H_2_O_2_/induced group are indicated as ^#^ *p* < 0.05, ^##^ *p* < 0.01, ^###^ *p* < 0.001; ^*^ *p* < 0.05, ^**^ *p* < 0.01, ^***^ *p* < 0.001 compared with the H_2_O_2_/induced group; and ^&^ *p* < 0.05, ^&&^ *p* < 0.01, ^&&&^ *p* < 0.001.

Excessive reactive ROS are not only a direct cause of cell apoptosis but also serve as a triggering signal for the neuroinflammatory cascade. Excessive ROS induce overexpression of iNOS through activation of the NF‐κB pathway, thereby catalyzing the production of the ONOO^−^ and forming a positive feedback loop of “oxidative stress–inflammation amplification” (Figure 5a) [29, 30, 31]. To verify the in vitro regulatory effect of PBB@AHA on the oxidative stress–inflammation axis, this study first quantitatively evaluated the intracellular ROS scavenging capacity of AHA, PBB, and PBB@AHA using the 2,7‐dichlorodihydrofluorescein diacetate (DCFH‐DA) probe. As shown in Figure 5b, H_2_O_2_ stimulation significantly increased ROS levels in PC12 cells. The AHA treatment group only slightly reduced ROS levels, whereas the PBB and PBB@AHA treatment groups decreased them by 58.3 ± 2.3% and 86.5 ± 1.4%, respectively. Notably, the ROS scavenging efficiency of the PBB@AHA treatment group was 48.4 ± 3.2% higher than that of PBB (Figure 5c), indicating that AHA modification enhances the cellular internalization of PBB and synergistically interacts with PBB to improve antioxidant capacity. Flow cytometry‐based quantitative analysis further confirmed that PBB@AHA significantly inhibits H_2_O_2_‐induced oxidative stress, with a strong positive correlation between ROS downregulation and reduced apoptosis rate (Figure 5d). To further investigate the regulatory effect of PBB@AHA on downstream inflammatory pathways of the “oxidative stress–inflammation axis”, we established a macrophage activation model induced by lipopolysaccharide (LPS)/interferon‐γ (IFN‐γ) to simulate the neuroinflammatory microenvironment. Since iNOS is the key enzyme that catalyzes the conversion of L‐arginine to NO, and NO is rapidly oxidized into the stable end product nitrite (NO_2_^−^) in the cell culture system, the nitrite content measured by the Griess method directly reflects iNOS functional activity. As shown in Figure 5e, PBB@AHA effectively inhibited iNOS activity, reducing nitrite production by 76.3 ± 3.1%. Its inhibitory efficiency was significantly superior to that of the individual components AHA (38.2 ± 2.1%) and PBB (54.7 ± 3.2%). As a key toxic product generated from the reaction between iNOS‐mediated NO and superoxide anion, the regulation of ONOO^−^ formation is a core aspect of understanding this pathological pathway. In this study, the ONOO^−^‐specific fluorescent probe hydroxyphenyl fluorescein (HPF) was used to directly detect the effect of PBB@AHA on its formation; in the LPS/IFN‐γ‐induced cell model, the HPF fluorescence intensity in the PBB@AHA treatment group was significantly reduced (Figure S13), confirming that it can efficiently inhibit ONOO^−^ formation.

Combining the above experimental results with the previously established vicious cycle mechanism of oxidative stress–iNOS cross‐talk, it can be concluded that PBB@AHA effectively scavenges ROS within the mitochondrial microenvironment. By inhibiting NF‐κB activation, it downregulates the iNOS‐NO signaling pathway, thereby preventing the formation of ONOO^−^. This enables targeted modulation of the “ROS‐iNOS‐ONOO^−^” axis, thereby alleviating oxidative damage to neuronal mitochondria, suppressing apoptosis, and offering a promising therapeutic strategy for ischemic stroke [32].

### 2.6 Verification of the In Vivo Therapeutic Effect and Biosafety of PBB@AHA

**FIGURE 6:**
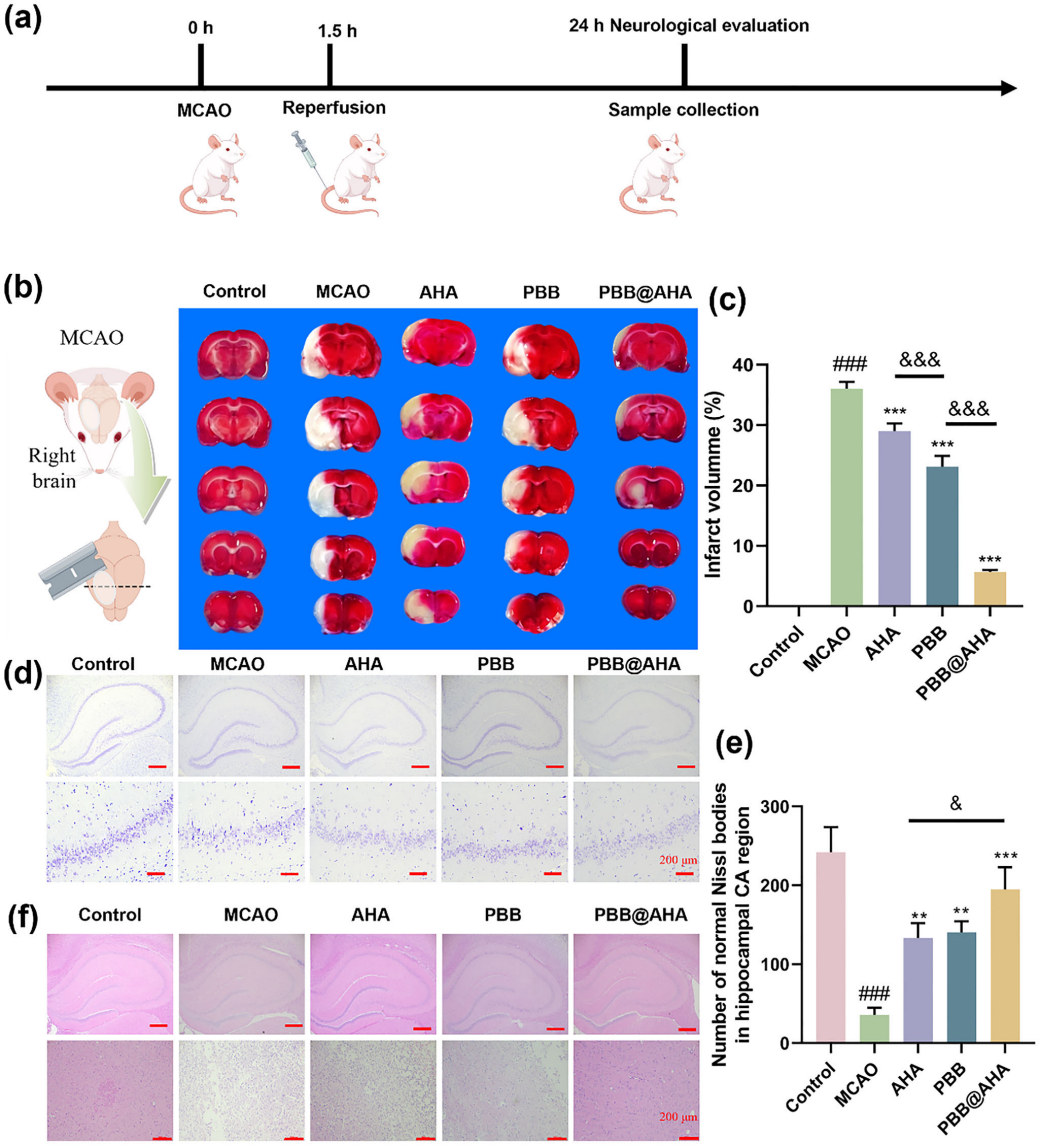
In vivo evaluation of the neuroprotective effect of PBB@AHA in ischemic stroke. (a) Schematic diagram illustrating PBB@AHA‐mediated brain protection in the MCAO rat model. (b) TTC‐stained brain sections from different treatment groups. (c) Quantitative comparison of infarct volumes across treatment groups (*n* = 3). (d) Nissl‐stained sections from various treatment groups (scale bar = 200 µm). (e) Statistical analysis of normal Nissl body counts (*n* = 3). (f) H&E‐stained sections from different treatment groups (scale bar = 200 µm) Significant differences between the control group and the MCAO model group are indicated as ^#^ *p* < 0.05, ^##^ *p* < 0.01, ^###^ *p* < 0.001; ^*^ *p* < 0.05, ^**^ *p* < 0.01, ^***^ *p* < 0.001 compared with the MCAO group; and ^&^ *p* < 0.05, ^&&^ *p* < 0.01, ^&&&^ *p* < 0.001.

Based on the regulatory mechanism of the oxidative stress–inflammation axis revealed in cell experiments, the therapeutic efficacy of PBB@AHA was systematically evaluated in a complex in vivo pathological environment by establishing a middle cerebral artery occlusion (MCAO) model in rats. Each group received intravenous administration of the respective therapeutic agent via the tail vein 1.5 h after MCAO‐induced ischemia, followed by 24 h of reperfusion. All rats underwent neurobehavioral assessment. Subsequently, the animals were euthanized and brain tissues were collected for further analysis (Figure 6a). Brain sections were stained with 2,3,5‐triphenyltetrazolium chloride (TTC) to assess infarct volume. Healthy brain tissue was stained red, whereas the infarcted regions remained unstained and appeared white. As shown in Figure 6b,c, brain sections from the control group exhibited uniform red staining, while the MCAO model group displayed distinct white infarct areas. Following 24 h of treatment, all intervention groups showed some degree of recovery; however, the PBB@AHA treatment group exhibited the most pronounced reduction in infarct volume (81.7 ± 3.3%), thereby preliminarily confirming its overall neuroprotective effect.

The morphological changes of neurons and the number of Nissl bodies following ischemic stroke are key histopathological indicators for assessing the extent of brain injury. Nissl staining was performed to label the Nissl bodies in neurons within the cerebral cortex and hippocampal CA regions, thereby evaluating the neuronal reparative capacity of PBB@AHA after ischemia‐reperfusion injury. As shown in Figure 6d,e, neurons in the cerebral cortex and hippocampal CA regions of the control group exhibited a plump morphology, with dark blue‐stained Nissl bodies clearly visible in the cytoplasm. In contrast, neurons from the MCAO group displayed marked shrinkage and deformation, with non‐selective staining due to cell membrane rupture. In the AHA and PBB treatment groups, partial morphological recovery of neurons was observed, whereas in the PBB@AHA group, most neurons retained a morphology closely resembling that of the control group, indicating superior neuroprotection. Furthermore, H&E staining (Figure 6f) revealed significant cellular atrophy (including cytoplasmic shrinkage and nuclear pyknosis) in the ischemic hemisphere of untreated MCAO rats, while all treatment groups showed varying degrees of morphological improvement [33]. Notably, the PBB@AHA group exhibited nearly normal neuronal architecture, further supporting its robust neuroprotective efficacy. Additionally, H&E staining of major organs demonstrated no signs of hepatic or renal toxicity, nor any histopathological abnormalities in the PBB@AHA treatment group (Figure S14), confirming its favorable safety profile and providing critical support for its potential clinical translation.

### 2.7 PBB@AHA Alleviates Neuronal Apoptosis After Ischemic Stroke

Neuronal apoptosis is recognized as a distinct feature in the pathological progression of neuronal death. Suppressing neuronal apoptosis has been shown to mitigate brain damage induced by ischemia. In this study, neuronal apoptosis was quantitatively assessed using NeuN/TUNEL staining, with fluorescence images analyzed and quantified via ImageJ software. The extent of neuronal apoptosis in the cerebral cortex (a region involved in sensory and motor functions) and the hippocampal CA area (a region associated with cognitive memory) was specifically examined to evaluate the neuroprotective efficacy of PBB@AHA in critical functional brain regions.

**FIGURE 7:**
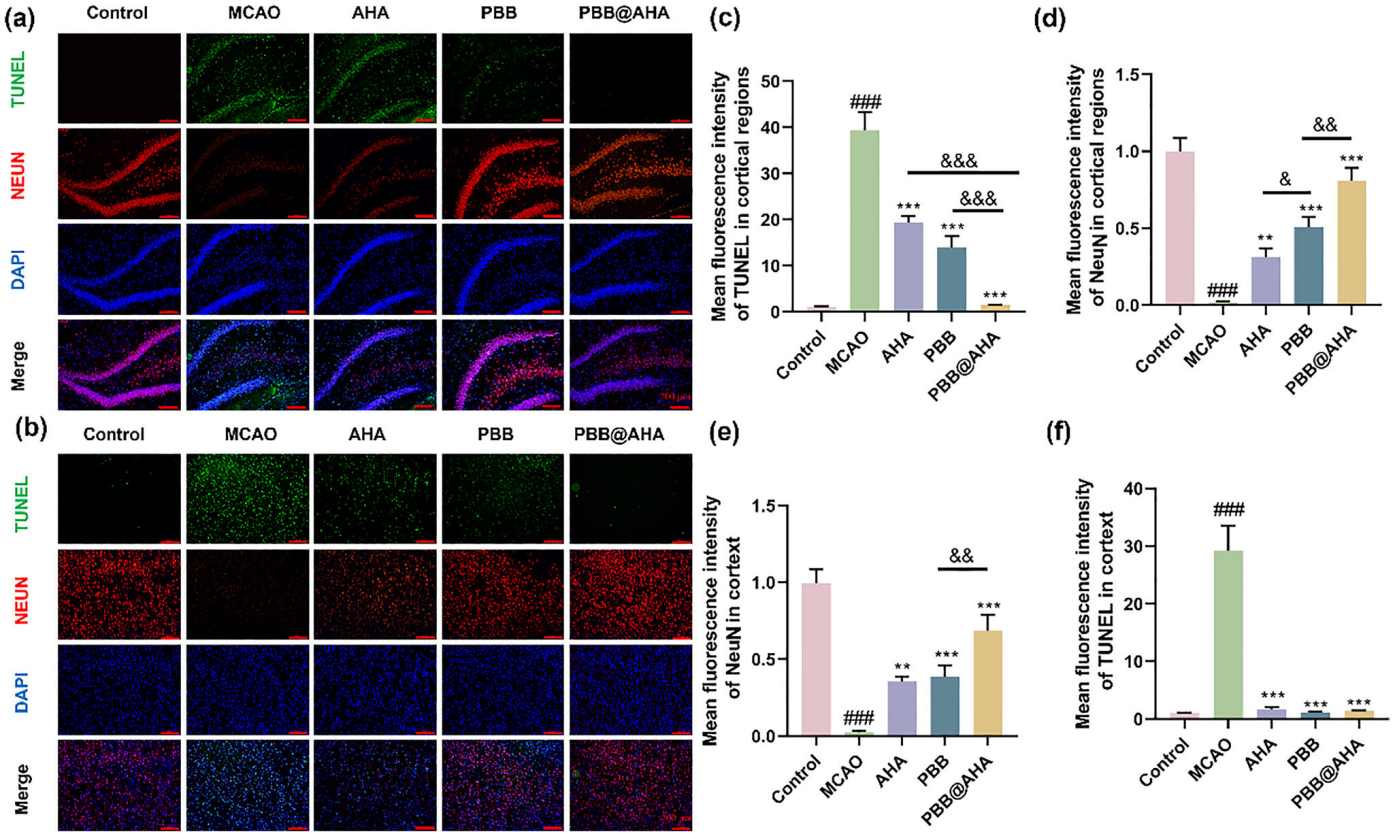
PBB@AHA suppresses ischemia stroke‐induced apoptosis and neuroinflammatory responses. (a) TUNEL staining (green) detecting apoptotic cells in the cerebral cortex. Neurons are labeled with NeuN (red), and cell nuclei are counterstained with DAPI (blue) (scale bar = 200 µm). (b) TUNEL staining (green) of apoptotic cells in the hippocampal CA area. Neurons are labeled with NeuN (red), and nuclei are stained with DAPI (blue) (scale bar = 200 µm). (c) Quantitative analysis of relative TUNEL fluorescence intensity in the cerebral cortex as an indicator of neuronal loss (*n* = 4). (d) Quantitative assessment of the percentage of NeuN‐positive neurons in the cerebral cortex (*n* = 4). (e) Quantitative evaluation of NeuN fluorescence intensity in the hippocampal CA area for assessing neuronal preservation (*n* = 4). (f) Quantitative measurement of the proportion of TUNEL‐positive neurons in the hippocampal CA area (*n* = 4). Significant differences between the control group and the MCAO model group are indicated as ^#^ *p* < 0.05, ^##^ *p* < 0.01, ^###^ *p* < 0.001; ^*^ *p* < 0.05, ^**^ *p* < 0.01, ^***^ *p* < 0.001 compared with the MCAO group; and ^&^ *p* < 0.05, ^&&^ *p* < 0.01, ^&&&^ *p* < 0.001.

The results demonstrated (Figure 7a–d): In the control group, neurons in the cerebral cortex exhibited intact morphology with no evident signs of apoptosis. In contrast, the MCAO model group showed a significant increase in TUNEL‐positive neurons, with an apoptosis rate reaching 53.4 ± 3.2%. All treatment groups improved neuronal survival and reduced apoptotic levels to varying degrees. Although AHA alone could partially inhibit apoptosis, its effect was relatively modest. Following treatment with PBB and PBB@AHA, the inhibition of neuronal apoptosis was markedly enhanced, with PBB@AHA showing the most pronounced effect, as evidenced by a substantial decrease in the number of TUNEL‐positive cells. The findings in the hippocampal CA area were consistent with those observed in the cerebral cortex (Figure 7e,f): In the MCAO group, hippocampal neuronal apoptosis was prominent, with approximately 61.2 ± 4.1% of neurons being TUNEL‐positive. Treatment with AHA and PBB reduced the apoptosis rates to 33.7 ± 2.3% and 43.2 ± 2.1%, respectively, preserving some hippocampal neurons. Notably, the PBB@AHA group exhibited the strongest anti‐apoptotic effect in the hippocampal CA area, suppressing neuronal apoptosis by up to 86.3 ± 3.3%. These findings indicate that PBB@AHA exerts a robust anti‐apoptotic effect in the MCAO rat model. Both the cerebral cortex and hippocampal CA area showed significantly reduced neuronal apoptosis after PBB@AHA treatment, outperforming either AHA or PBB monotherapy. This observation is highly consistent with the results from the H_2_O_2_‐induced apoptosis assay in PC12 cells.

### 2.8 PBB@AHA Inhibits the Oxidative Stress–Inflammation Axis After Ischemic Stroke

**FIGURE 8:**
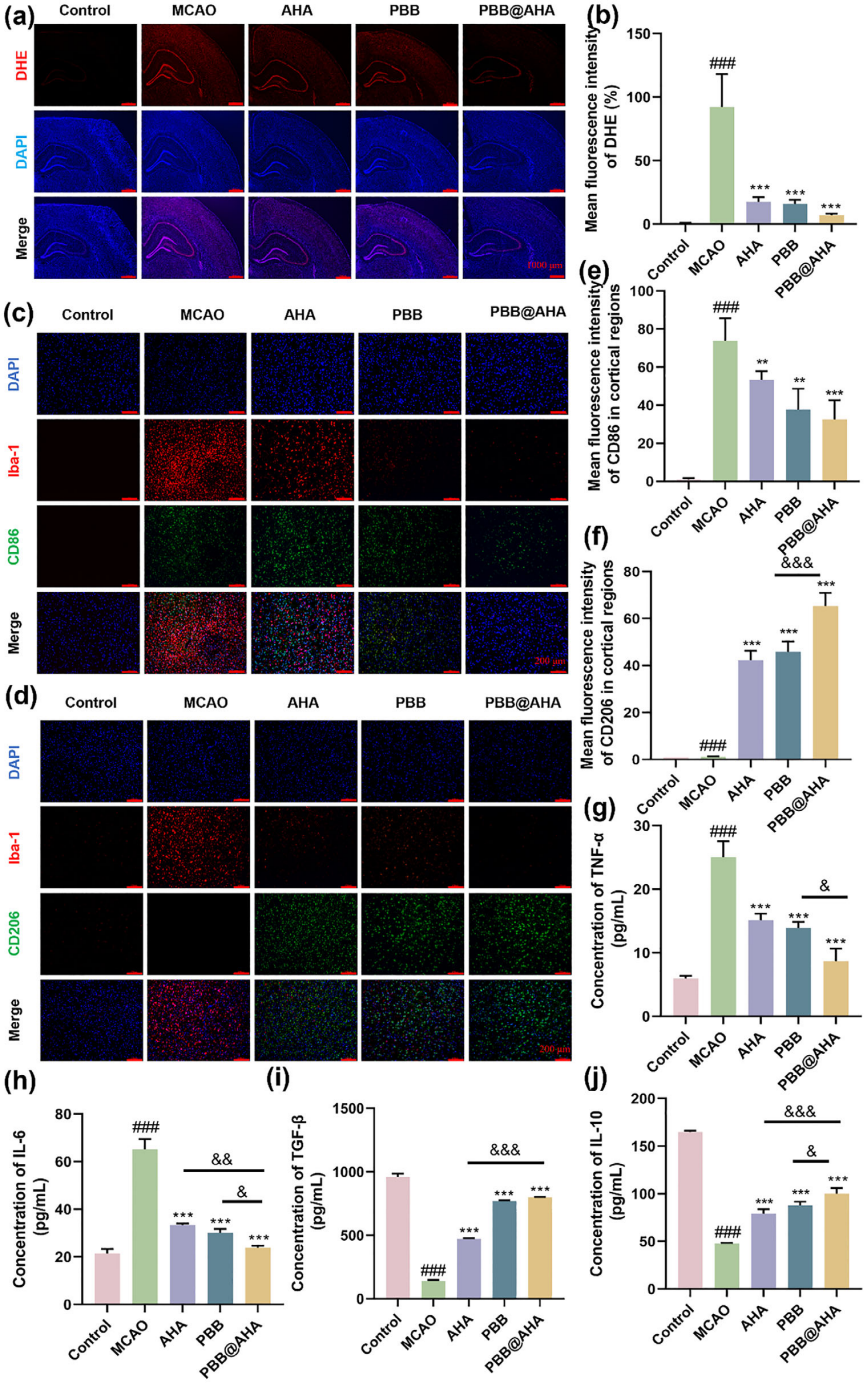
PBB@AHA regulates brain inflammation in MCAO rats by modulating the phenotype of neuroglial cells. (a) DHE staining images in the cerebral cortex of each treatment group (scale bar = 1000 µm). (b) Quantitative analysis of DHE fluorescence intensity in brain tissue. (c) Iba‐1 staining (red) showing immunologically activated microglia in the cerebral cortex. Cell nuclei were labeled with DAPI (blue), and CD86 was used as a marker for M1 microglia (green, *n* = 4, scale bar = 200 µm). (d) Iba‐1 staining (red) showing immunologically activated microglia in the cerebral cortex. Cell nuclei were labeled with DAPI (blue), and CD206 was used as a marker for M2 microglia (green, *n* = 4, scale bar = 200 µm). (e) Quantitative statistical results of average CD86 fluorescence intensity in the cerebral cortex. (f) Quantitative statistical results of average CD206 fluorescence intensity in the cerebral cortex. (g–j) Levels of inflammatory cytokines TNF‐α (g), IL‐6 (h), TGF‐β (i), and IL‐10 (j) in the infarcted brain region following different treatments (*n* = 4). Significant differences between the control group and the MCAO model group are indicated as ^#^ *p* < 0.05, ^##^ *p* < 0.01, ^###^ *p* < 0.001; ^*^ *p* < 0.05, ^**^ *p* < 0.01, ^***^ *p* < 0.001 compared with the MCAO group; and ^&^ *p* < 0.05, ^&&^ *p* < 0.01, ^&&&^ *p* < 0.001.

To elucidate the neuroprotective mechanism of PBB@AHA, this study systematically evaluated its effects from two key perspectives: oxidative stress regulation and immune microenvironment remodeling. First, the dihydroethidium (DHE) fluorescent probe was employed to assess ROS levels in the rat brain following cerebral ischemia (Figure 8a,b). The results revealed a significantly elevated red fluorescent signal in the MCAO model group, whereas the PBB@AHA treatment reduced ROS intensity to 12.3 ± 1.2% of that observed in the MCAO group. This indicates that PBB@AHA effectively scavenges reactive oxygen species and interrupts the oxidative damage cascade. Notably, there exists a significant synergistic relationship between ROS clearance and neuroimmune microenvironment modulation. Microglia and astrocytes, as primary immune effector cells in the central nervous system, play a pivotal role in determining the progression of brain injury. Excessive inflammatory responses after stroke can exacerbate neuronal damage by promoting M1 polarization of microglia. Conversely, inducing the transition of microglia from the pro‐inflammatory M1 phenotype to the anti‐inflammatory M2 phenotype enables concurrent alleviation of oxidative stress and suppression of neuroinflammation [34]. Here, this study established an MCAO rat model and employed immunohistochemistry to assess the expression levels of glial fibrillary acidic protein (GFAP, a marker of activated astrocytes), ionized calcium‐binding adapter molecule 1 (Iba‐1, a marker of microglia), CD86 (a marker of M1 microglia), and CD206 (a marker of M2 microglia) in brain sections from different treatment groups (Figure 8c,d, Figure S15). In brain sections from MCAO rats administered only physiological saline, the expression levels of Iba‐1 and GFAP were markedly elevated, indicating that the MCAO model induced immune activation of resting microglia and astrocytes. Following 24 h treatment with various therapeutic agents, the number of Iba‐1‐positive cells decreased relatively, with PBB@AHA demonstrating the strongest inhibitory effect on microglial activation. Meanwhile, compared with the MCAO group, PBB@AHA treatment reduced the expression of the M1 microglia marker CD86 by 57.2 ± 1.3% and increased the expression of the M2 microglia marker CD206 by 63.1 ± 2.2% (Figure 8e,f). Moreover, PBB@AHA exhibited superior regulatory effects compared to its individual components (CD86 inhibition: AHA group 29.8%; PBB group 50.4%. CD206 upregulation: AHA group 41.8 ± 1.8%; PBB group 43.2 ± 2.1%). The expression level of iNOS, a key pro‐inflammatory enzyme involved in mediating the inflammatory cascade, is closely correlated with the severity of neuroinflammation. Immunofluorescence analysis of iNOS in the ischemic cortex of MCAO rats (Figure S16) revealed that PBB@AHA significantly reduced iNOS expression by 68.4 ± 1.3% compared to the MCAO group, outperforming both the AHA group (62.9 ± 2.6%) and the PBB group (44.9 ± 4.1%). This finding was consistent with the Griess assay results, further supporting the role of PBB@AHA in modulating the inflammatory response through iNOS inhibition. Additionally, we used the enzyme‐linked immunosorbent assay (ELISA) to evaluate the secretion of pro‐inflammatory and anti‐inflammatory cytokines in the infarcted regions of MCAO rat brains [35]. The results (Figure 8g,h) showed that in the MCAO model group, the levels of pro‐inflammatory cytokines TNF‐α and IL‐6 increased to 4.2‐fold and 3.1‐fold those of the control group, respectively. After PBB@AHA intervention, TNF‐α and IL‐6 levels decreased by 2.8‐fold and 3.1‐fold, respectively. Simultaneously, the levels of anti‐inflammatory cytokines IL‐10 and TGF‐β increased to 2.1‐fold and 5.7‐fold those of the MCAO group, respectively. Collectively, this evidence supports that PBB@AHA exerts its neuroprotective effects via dual‐mechanism synergy. On one hand, it scavenges ROS to preserve neuronal mitochondrial integrity. On the other hand, it remodels the neuroimmune microenvironment by suppressing the activation of Iba‐1‐positive microglia and GFAP‐positive astrocytes, promoting microglial polarization toward the M2 phenotype, facilitating astrocytic quiescence, and restoring the dynamic balance between pro‐inflammatory and anti‐inflammatory factors‐thereby establishing a favorable microenvironment conducive to neural repair.

## 3 Discussion

IS pathological progression is regulated by a cascade of oxidative stress, inflammatory responses, and apoptosis, accompanied by critical therapeutic bottlenecks including low BBB permeability (drug penetration rate < 2%) and absence of lesion‐specific drug release. Cerebral ischemia‐reperfusion injury triggers mitochondrial dysfunction, explosive accumulation of ROS, and intracellular acidosis (pH < 6.5), which subsequently activates iNOS‐mediated overproduction of NO. The resulting ONOO^−^ exacerbates neuroinflammation, promotes ΔΨm collapse, and accelerates neuronal apoptosis, ultimately forming a self‐sustaining pathological cycle. Single‐target therapies (e.g., ROS scavenger PBB, iNOS inhibitor N3) fail to interrupt this cascade reaction, while the BBB penetration capacity of most drugs remains severely constrained due to inadequate lipophilicity.

Here, we developed a pH/ROS dual‐responsive lipid‐nanozyme system (PBB@AHA) via dynamic covalent bond assembly, integrating “BBB penetration enhancement, microenvironment‐responsive release, and multi‐target synergy.” Inspired by phospholipid structures, we linked N3, PCA, and VES‐APBA (vitamin E‐derived lipid derivative) via pH‐sensitive imine bonds and ROS‐sensitive borate ester bonds, forming a dynamic lipid module (AHA) that self‐assembled with PBB through electrostatic interactions. VES‐APBA's lipid properties enable BBB crossing via “passive diffusion (lipid raft‐mediated) + active transport (energy‐dependent)” synergy, achieving a penetration rate of 35.2 ± 3.2%—2.3‐fold higher than unmodified PBB (Figure 3b). In vivo IVIS imaging showed a 5.8:1 fluorescence intensity ratio of ischemic to contralateral hemispheres at 6 h post‐injection, confirming lesion‐specific enrichment and prolonged retention (12 h) (Figure 3e). Mechanistic studies revealed PBB@AHA penetrates via the “lipid raft‐Caveolin‐1 pathway,” avoiding lysosomal degradation and distinguishing from PBB's “clathrin‐dependent endocytosis” (Figure 3f–i).

Under the ischemic microenvironment, dual‐responsive bonds in PBB@AHA cleave, synchronously releasing PBB, N3, and PCA. In vitro assays confirmed PBB@AHA mimics SOD/CAT dual‐enzyme activity, scavenging 73.2% of •OH and 53% of O_2_^•−^, with total ROS clearance 48.4% higher than PBB alone (Figure 2b–e). It restored ΔΨm of H_2_O_2_‐induced PC12 cells to 87.1%‐superior to AHA (20.3%) and PBB (48.1%)—and reduced apoptosis rate to 6.8 ± 0.9% (Figure 4e,f). In LPS/IFN‐γ‐induced RAW264.7 cells, PBB@AHA inhibited iNOS activity by 76.3%, blocking ONOO^−^ formation more effectively than single components (Figure 5e), thus targeting the “ROS‐iNOS‐ONOO^−^” axis.

In vivo experiments using a rat MCAO model verified PBB@AHA's efficacy: it reduced infarct volume by 81.7 ± 3.3% and improved neurological function scores by 73.2 ± 4.1% (Figure 6b,c). Nissl and H&E staining confirmed preserved neuronal morphology in the cerebral cortex and hippocampal CA region (Figure 6d–f), while NeuN/TUNEL staining showed 86.3% inhibition of hippocampal neuronal apoptosis (Figure 7a–f). PBB@AHA reshaped the neuroimmune microenvironment: it reduced ROS levels to 12.3% of the MCAO group (Figure 8a,b), downregulated M1 marker CD86 by 57.2%, upregulated M2 marker CD206 by 63.1% (Figure 8c–f), and rebalanced inflammatory factors (TNF‐α/IL‐6 reduced by 2.8/3.1 folds; IL‐10/TGF‐β increased by 2.1/5.7 folds) (Figure 8g–j).

Compared with existing therapies, PBB@AHA offers distinct advantages: it avoids complex modification of targeted nanoparticles, overcomes low penetration of traditional lipid carriers, and surpasses single‐component nanozymes via multi‐target synergy. Unlike cell membrane‐coated nanozymes (poor stability), PBB@AHA uses simple electrostatic self‐assembly and structural biomimicry, enhancing scalability for clinical translation.

Despite these findings, limitations remain. The H_2_O_2_‐induced in vitro model cannot fully recapitulate cerebral ischemia‐reperfusion complexity; future studies will adopt the OGD/R model for clinically relevant validation. Long‐term biosafety and metabolic pathways require systematic evaluation via PET‐CT and metabolomics. Additionally, the molecular pathways mediating PBB@AHA‐induced M2 microglia polarization (e.g., NF‐κB, STAT6) need further dissection using multi‐omics approaches.

In summary, PBB@AHA addresses IS bottlenecks via phospholipid‐mimetic BBB penetration, dual‐responsive lesion‐specific release, and multi‐target cascade inhibition. This work provides a precision therapeutic platform for IS and new insights for complex multi‐target diseases. Future research will optimize models, evaluate long‐term safety, and elucidate molecular mechanisms to accelerate clinical translation.

## 4 Conclusions

Based on the pathological microenvironment‐responsive design concept, this study developed a pH/ROS dual‐responsive PBB@AHA co‐delivery system, providing an innovative therapeutic strategy for ischemic stroke. The system was constructed using a biomimetic phospholipid double‐tail architecture, where the iNOS inhibitor N3, neuroprotective agent PCA, and VES‐APBA were conjugated via pH‐sensitive imine bonds and ROS‐sensitive borate ester bonds to form a dynamic lipid module. This module then underwent electrostatic self‐assembly with PBB to establish a dual‐responsive PBB@AHA system capable of multi‐target synergy and efficient enrichment. Leveraging its lipid characteristics, VES‐APBA efficiently crosses the BBB through both passive diffusion and active transport, accumulating specifically at the lesion site. Under the characteristic low pH and high ROS conditions of the ischemic microenvironment, the dual‐responsive bonds cleave, enabling synchronized co‐release of N3, PCA, and PBB. At the cellular level, PBB scavenges reactive oxygen species and maintains ΔΨm stability, while AHA downregulates iNOS expression to reduce ONOO^−^ generation. Together, they synergistically inhibit apoptosis and inflammatory responses. Animal experiments demonstrated that this system reduces activated astrocyte density, promotes their transition to a quiescent state, induces M2 polarization of microglia, reestablishes pro‐/anti‐inflammatory cytokine balance, and remodels the neural microenvironment. By overcoming the limitations of single‐function nanomedicines, it achieves integrated intervention through molecular repair, organelle protection, and neuroimmune regulation, effectively blocking the oxidative stress–inflammation–apoptosis cascade via multi‐level regulation and offering a novel pathology‐driven precision therapeutic approach for ischemic stroke. Future work will focus on elucidating the epigenetic mechanisms underlying glial cell phenotypic switching and exploring integrated applications of neurotrophic delivery systems to refine the treatment strategy.

## 5 Experimental Section/Methods

### 5.1 Materials

1,3‐Diaminoguanidine hydrochloride (98%), lipoic acid (99%), N,N′‐disuccinimidyl carbonate (99%), simvastatin (≥ 98%), α‐linolenic acid (i.e., omega‐3), polyvinylpyrrolidone K30 (PVP), potassium ferricyanide trihydrate (K_3_Fe(CN)_6_·3H_2_O), 3‐aminophenylboronic acid (≥ 98%), vitamin E (≥ 99%), protocatechualdehyde (PCA, 99%), N‐hydroxysuccinimide (NHS), 1‐ethyl‐3‐(3‐dimethylaminopropyl)carbodiimide (EDC), 4′,6‐diamidino‐2‐phenylindole dihydrochloride (DAPI, Cat. No. 44946B, 98%), 2,3,5‐triphenyltetrazolium chloride (TTC, ≥ 98%), and 2,2′‐azino‐bis(3‐ethylbenzothiazoline‐6‐sulfonic acid) (ABTS) were obtained from Titan S (China). 2′,7′‐Dichlorodihydrofluorescein diacetate (DCFH‐DA) fluorescent probe (Cat. No. C9075, ≥ 97%), 5,5′,6,6′‐tetrachloro‐1,1′,3,3′‐tetraethylbenzimidazolylcarbocyanine iodide (JC‐1) mitochondrial membrane potential assay kit (Cat. No. C8134), hydroxyphenyl fluorescein (HPF), 3‐(4,5‐dimethylthiazol‐2‐yl)‐2,5‐diphenyltetrazolium bromide (MTT), Calcein‐AM dye, propidium iodide (PI), 2,2‐diphenyl‐1‐picrylhydrazyl (DPPH), nitroblue tetrazolium (NBT), riboflavin, methionine, and Griess reagent were purchased from Adamas (China). Annexin V/FITC apoptosis detection kit (Cat. No. C1383M), Hoechst staining solution, Nile Red, rhodamine B (RhB), rat interleukin‐6 (IL‐6) enzyme‐linked immunosorbent assay (ELISA) kit (Cat. No. PI328), rat tumor necrosis factor‐alpha (TNF‐α) ELISA kit (Cat. No. PT516), rat interleukin‐10 (IL‐10) ELISA kit (Cat. No. PI525), mouse/rat transforming growth factor‐beta 1 (TGF‐β1) ELISA kit (Cat. No. PT878), and bicinchoninic acid (BCA) protein assay kit were acquired from Beyotime Biotechnology (China). Superoxide dismutase (SOD) activity assay kit (Cat. No. BC5165), catalase (CAT) activity assay kit (Cat. No. BC0205), cluster of differentiation 86 (CD86) antibody (Cat. No. K010082P, dilution ratio: 1:500), and cluster of differentiation 206 (CD206) antibody (Cat. No. K011692M, dilution ratio: 1:4000) were obtained from Solarbio Life Sciences (China). Silicon‐coated nylon thread (Cat. No. A5‐283850) was purchased from Beijing Xinong Technology Co., Ltd. (China). Iba‐1 antibody (Cat. No. 81728‐1‐RR, dilution ratio: 1:1000) was obtained from Proteintech Group, Inc. Neuronal nuclei (NeuN) antibody, Alexa Fluor 647‐conjugated secondary antibody, and fluorescein isothiocyanate (FITC)‐conjugated secondary antibody were all purchased from Abcam plc. (USA).

### 5.2 Characterization

Comprehensive characterization was performed through various microscopic and spectral analyses. Specifically, a Malvern particle size analyzer was used to determine particle size distribution and Zeta potential, while morphology and elemental composition were assessed using TEM (JEOL JEM‐F200, 100 kV, Japan), high‐resolution transmission electron microscopy (HRTEM) (JEOL 2010), and energy‐dispersive spectroscopy (EDS). Structural information and surface valence distribution of PBB were obtained through XRD (Bruker D2 Phaser, Germany).

### 5.3 Statistical Analysis

All experiments were independently repeated at least three times, and data were expressed as mean and standard deviation. GraphPad Prism 9 (GraphPad Software, Inc., La Jolla, CA, USA) was applied for data analysis and comparison. Statistical comparisons between two groups were performed using a two‐tailed Student's t‐test. Statistical comparisons between multiple groups (more than two groups) were performed using one‐way analysis of variance (ANOVA) and Tukey's post hoc test. ^*^ *p* < 0.05, ^**^ *p* < 0.01, and ^***^ *p* < 0.001 were considered statistically significant differences.

## Author Contributions

**Mengcheng Guo**: writing – original draft, visualization, methodology, investigation, formal analysis, and conceptualization. **Yapeng Li** and **Meili Shen**: writing – review & editing, project administration, funding acquisition, and conceptualization. **Qingran Guan**: validation, methodology, investigation, and formal analysis. **Zhen Li**: visualization, software, investigation. **Lixue Zhang**: supervision and resources. **Man Liu**: supervision and resources. **Guanyu Qiao**: resources, data curation. **Qingbiao Yang**: resources and formal analysis. **Linlin Liu**: data curation and conceptualization.

## Ethics Statement

All animal experiments were performed under the guidance of the Animal Ethics and Experiment Committee of Jilin University. All animal experiments were approved by the Animal Ethics and Experiment Committee of Jilin University (approval number:2024 extension No. 552).

## Conflicts of Interest

The authors declare no conflicts of interest.

## Supporting information




**Supporting File**: advs75180‐sup‐0001‐SuppMat.docx.

## Data Availability

The data that support the findings of this study are available from the corresponding author upon reasonable request.
