# Supplementary material for: Dual‐Responsive Dynamic Covalent Bond‐Based Assembly of Lipid‐Nanozyme Systems via Multi‐Target Synergy and Efficient Target Enrichment for Ischemic Stroke Therapy
Source: Adv Sci (Weinh). 2026 Apr 7:e19226. Online ahead of print. doi: 10.1002/advs.202519226 (PMC13334673; doi:10.1002/advs.202519226)
Supplement: Supplementary file 1 — Supporting File: advs75180‐sup‐0001‐SuppMat.docx. [file ADVS-9999-e19226-s001.docx]

Supporting Information

Dual-Responsive Dynamic Covalent Bond-Based Assembly of Lipid-Nanozyme Systems via Multi-Target Synergy and Efficient Target Enrichment for Ischemic Stroke Therapy

Mengcheng Guo^a,1^, Qingran Guan^a,1^, Guanyu Qiao^b^, Lixue Zhang^a^, Man Liu^a^, Zhen Li^a^, Qingbiao Yang^c^, Meili Shen*^b^, Linlin Liu*^b^, Yapeng Li*^a^

^a^ Engineering Research Center of High Performance Plastics, Ministry of Education, College of Chemistry, Jilin University, Changchun, Jilin, 130012, China.

^b^ Department of Radiation Oncology, China-Japan Union Hospital of Jilin University, Changchun, Jilin, 130033, China.

^c^ College of Chemistry, Jilin University, Changchun, Jilin, 130021, China.

Corresponding authors: Linlin Liu, liulinl@jlu.edu.cn. Department of Radiation Oncology, China-Japan Union Hospital of Jilin University, Changchun, Jilin, 130033, China. Yapeng Li, liyapeng@jlu.edu.cn. Engineering Research Center of High Performance Plastics, Ministry of Education, College of Chemistry, Jilin University, Changchun, 130012, China. Meili Shen, shenmeili@jlu.edu.cn. Department of Radiation Oncology, China-Japan Union Hospital of Jilin University, Changchun, Jilin, 130033, China.

**Experimental Section/Methods**

**Synthesis of a series of phenylboronic acid-functionalized lipid derivatives (PBA-LD):** Synthesis of phenylboronic acid-modified VES (VES-APBA): A solution of triethylamine (0.35 mL, 2.5 mmol) was slowly added to a stirred solution of tocopherol (4.3 g, 10 mmol) and succinic anhydride (1.5 g, 15 mmol) in toluene (20 mL). The reaction mixture was then stirred at 60 ℃ for 5 hours to ensure complete reaction. Upon completion, the reaction was quenched by the addition of 10 mL of water, followed by extraction with dichloromethane (3 × 10 mL). The combined organic layers were sequentially washed with 1 mol/L hydrochloric acid (3 × 50 mL) and water (2 × 30 mL), dried over anhydrous sodium sulfate, and concentrated under reduced pressure to afford the crude product. The crude residue was then dissolved in a minimal amount of warm solvent and precipitated by the addition of 100 mL of ice-cold n-hexane, followed by crystallization at 4 ℃ for 26 hours to yield purified vitamin E succinate (VES) (Figure S1).

The carboxyl group of vitamin E succinate (VES, 1 mmol) was activated using N-hydroxysuccinimide (NHS, 2 mmol) and 1-ethyl-3-(3-dimethylaminopropyl) carbodiimide (EDC, 2 mmol) in 10 mL of ethanol at room temperature for 12 hours. After activation, the organic solvent was removed under reduced pressure, and the residue was washed three times with ice-cold water to eliminate residual NHS and EDC. The carboxyl-activated intermediate, VES-NHS, was then obtained by vacuum drying. Subsequently, VES-NHS (0.5 mmol, 629 g/mol, 315 mg) was dissolved in 10 mL of ethanol, followed by the addition of 3-aminophenylboronic acid (5 mmol, 371.96 g/mol, 1.8598 g), and the reaction was allowed to proceed at room temperature for 12 hours. The resulting product was purified by washing with ice-cold water and identified as VES-APBA (Figure S2).

**Synthesis of lipoic acid phenylboronic acid (LA-APBA):** 4.8 g of lipoic acid (LA) and 7.2 g of N, N’-disuccinimidyl carbonate were dissolved in 200 mL of acetonitrile and stirred to ensure complete mixing. Triethylamine (10 mL) was then added, and the reaction mixture was stirred at room temperature for 2 hours. Subsequently, two-thirds of the solvent was removed under reduced pressure, and the remaining solution was poured into 275 mL of saturated sodium bicarbonate solution. A yellow precipitate formed, which was collected by filtration and dissolved in dichloromethane. The resulting clear yellow solution was concentrated under vacuum to afford the carboxyl-activated intermediate, LA-NHS (Figure S3). Next, LA-NHS (0.5 mmol) was dissolved in 10 mL of tetrahydrofuran (THF), followed by the addition of 3-aminophenylboronic acid (5 mmol, 371.96 g/mol, 1.8598 g). The reaction mixture was stirred in the dark at room temperature for 24 hours to ensure complete coupling. After completion, the THF was removed by rotary evaporation, and the residue was subjected to extraction with dichloromethane and saturated brine (3 × each phase) to fully separate the organic layer. The organic phase was dried over anhydrous sodium sulfate and concentrated under vacuum to yield the target product, LA-APBA (Figure S4).

**Synthesis of simvastatin acid phenylboronic acid (SA-APBA):** In a 100 mL single-necked flask, 1 g of simvastatin was dissolved in 10 mL of ethanol under continuous stirring until complete dissolution. Subsequently, 45 mL of 0.1 M sodium hydroxide solution was added, and the reaction mixture was stirred at 50 ℃ for 2 hours. Upon completion, the pH of the reaction solution was adjusted to neutrality using hydrochloric acid. Ethanol was removed under reduced pressure using a rotary evaporator, followed by extraction with n-butanol. Simvastatin acid was obtained after further rotary evaporation and vacuum drying. The carboxyl group of simvastatin acid (SA, 1 mmol) was activated with N-hydroxysuccinimide (NHS, 2 mmol) and 1-ethyl-3-(3-dimethylaminopropyl) carbodiimide (EDC, 2 mmol) in 10 mL of ethanol at room temperature for 2 hours. Then, 3-aminophenylboronic acid (5 mmol, 371.96 g/mol, 1.8598 g) was added, and the reaction was allowed to proceed at room temperature for 12 hours. The reaction mixture was extracted three times with dichloromethane and saturated brine. The organic phase was collected, dried over anhydrous sodium sulfate, and concentrated under vacuum to yield the target product SA-APBA (Figure S5).

**Synthesis of omega-3 phenylboronic acid:** The carboxyl group of omega-3 (1 mmol) was activated using N-hydroxysuccinimide (NHS, 2 mmol) and 1-ethyl-3-(3-dimethylaminopropyl) carbodiimide (EDC, 2 mmol) in 10 mL of ethanol at room temperature for 2 hours. Subsequently, 3-aminophenylboronic acid (5 mmol, 371.96 g/mol, 1.8598 g) was added, and the reaction was allowed to proceed at room temperature for 12 hours. The resulting product was purified by washing with ice-cold water and obtained as the target compound (Figure S6).

**Synthesis and assembly of dynamic liposomal prodrug molecules (An-AHA):** PBA-LD、PCA and N3 were dissolved in 990 μL of DMSO at a molar ratio of 1:1:0.5. Subsequently, an aqueous solution of 1,3-diaminoguanidine hydrochloride (100 mg/mL, 10 μL) was slowly added to the mixture. The reaction mixture was stirred at room temperature for 6 hours to obtain the crude reaction solution. Then, 50 μL of the reaction solution was added dropwise to 1 mL of phosphate-buffered saline (PBS, 10 mM, pH 7.4). After stirring for 2 hours, An-AHA (n = 1, 2, 3 or 4) was isolated via ultrafiltration (MWCO = 10 kDa).

**Synthesis of Prussian blue nanozyme (PBB):** K_3_Fe(CN)_6_·3H_2_O (696 mg) and polyvinylpyrrolidone K30 (PVP, 8 g) were added to 40 mL of 0.01 M HCl and stirred for 30 minutes. The mixture was then heated to 100 ℃ in a water bath and maintained under reflux for 24 hours. After cooling to room temperature, the resulting PBB nanoparticles were collected by centrifugation and washed multiple times. The purified product was freeze-dried and stored at 4 ℃ for subsequent experiments.

Assembly of PBB@AHA: A total of 200 μL of PBB solution (1 mg/mL) was added to 3.6 mL of PBS buffer solution and stirred to ensure uniform dispersion. Subsequently, 200 μL of AHA DMSO stock solution was added dropwise. Upon observation of turbidity in the initially clear blue solution, the mixture was stirred for 12 hours at room temperature under a nitrogen atmosphere and in the dark. The assembled PBB@AHA complex was then purified by ultrafiltration (MWCO = 10 kDa).

**Characterization:**

A comprehensive characterization was conducted using various microscopic and spectroscopic techniques. The molecular structure was confirmed by 400 MHz nuclear magnetic resonance (NMR), high-performance liquid chromatography-mass spectrometry (LC-MS), and Fourier-transform infrared spectroscopy (FT-IR). Particle size distribution and zeta potential were measured using a Malvern Zetasizer. Morphology and elemental composition were analyzed via high-resolution transmission electron microscopy (TEM, FEI Talos-F200X) and energy-dispersive X-ray spectroscopy (EDS). The crystal structure of the sample was determined by X-ray diffraction (XRD, Bruker D2 PHASER, Germany).

**Responsive Release:** To evaluate the stimuli-responsive drug release behavior of AHA, 1 mg of the sample was resuspended in 1 mL of four different phosphate-buffered saline (PBS) solutions: PBS (pH 7.4), PBS (pH 7.4, 100 μM H_2_O_2_), PBS (pH 6.5), and PBS (pH 6.5, 100 μM H_2_O_2_). The suspensions were incubated at 37 ℃ for 2 hours. Subsequently, the reaction mixtures were analyzed by high-performance liquid chromatography (HPLC) to monitor changes in the chromatographic peak profiles under varying conditions.

**Antioxidant Test:**

**Electron Paramagnetic Resonance (EPR):** The hydroxyl radical (•OH) scavenging capability was assessed using EPR analysis. •OH radicals were generated via the Fenton reaction using a Fe^2+^/H_2_O_2_ system composed of 1.8 mM FeSO_4_ and 5 mM H_2_O_2_. The sample (20 μg/mL) was introduced to evaluate its ability to neutralize •OH radicals.

**Free radical scavenging experiments:**

**DPPH radical scavenging assay:** A DPPH solution (125 μM) was mixed with varying concentrations of NPs at a 1:1 volume ratio and incubated at 37 ℃ for 15 minutes. The absorbance at 517 nm was measured using UV-Vis spectroscopy to determine the extent of DPPH• scavenging.

**Superoxide radical (O_2_^•−^) scavenging efficiency:** The scavenging efficiency of O_2_^•−^ was assessed by measuring the inhibition of nitroblue tetrazolium (NBT) photoreduction. A reaction mixture containing riboflavin (20 μM), methionine (12.5 mM), NBT (75 μM), and different concentrations of nanoparticles (50 μg/mL) was prepared in PBS. The mixture was exposed to UV light of constant intensity at 25 ℃ for 5 minutes. The negative control group contained riboflavin, methionine, and NBT without UV exposure, while the positive control group contained the same components but was exposed to UV light. The treated sample group included riboflavin, methionine, NBT, and NPs, and was also exposed to UV light. All experimental procedures were performed in the dark. The O_2_^•−^ scavenging rate was calculated using the following formula:

O_2_^•−^ scavenging effect (%) = (Ap − As) / (Ap − An) × 100%

where As, An, and Ap represent the absorbance values of the treated sample, negative control, and positive control, respectively.

**SOD and CAT-mimicking activity assay**

The SOD-mimicking activity of PBB@AHA was investigated through its O_2_^•−^ scavenging activity with the commercial SOD assay kit (BC5165, Solarbio, Beijing, China). The assay was performed according to the manufacturer’s instructions, and the SOD-mimicking specific activity was calculated using the provided standard curve and formula:

SOD Activity (U/mg) = (Sample Inhibition Rate / (1 - Sample Inhibition Rate)) × Dilution Factor / (Reaction Time × Sample Amount).

The CAT-mimicking activity of PBB@AHA was determined via H_2_O_2_ decomposition assay using the commercial CAT activity kit (BC0205, Solarbio, Beijing, China). The experiment was conducted following the manufacturer’s protocols, and the CAT-mimicking specific activity was quantified based on the kit’s standard calibration curve.

**Cells:**

Human umbilical vein endothelial cells (HBMEC) and PC12 neuronal cells were cultured in DMEM complete medium consisting of 89% basal medium, 10% fetal bovine serum (FBS), and 1% penicillin-streptomycin. The cells were maintained in a humidified incubator at 37 ℃ with 5% CO_2_.

**Cell uptake assay:**

Cellular uptake behavior was evaluated using confocal laser scanning microscopy (CLSM). In brief, PC12 cells were seeded in confocal dishes at a density of 1 × 10^5^ cells per well and incubated for 12 hours. Cellular stress was induced by adding H_2_O_2_ (600 μM), followed by an additional 12-hour incubation period. To assess cellular uptake, the cells were treated with DMEM complete medium containing varying concentrations of NPs for 4 hours, then washed three times with PBS. Hoechst staining was performed for 30 minutes to label the nuclei. The cells were subsequently observed at defined time points using a fluorescence microscope.

**Intracellular ROS scavenging:**

The intracellular reactive ROS level was assessed using the 2,7-dichlorofluorescein diacetate (DCFH-DA) fluorescent probe. PC12 cells were seeded in 6-well plates at a density of 1 × 10^5^ cells per well and cultured for 24 hours. Following H_2_O_2_ (600 μM) treatment to induce oxidative stress, the cells were exposed to 25 μg/mL of the sample in PBS for 5 hours under pH 7.4 conditions. After incubation, the culture medium was removed, and the cells were rinsed three times with PBS. Subsequently, the cells were incubated with 2 mL of DCFH-DA solution (15 μg/mL) for 15 minutes. After washing three times with PBS, the cells were visualized using a fluorescence microscope. Additionally, the cells were trypsinized, collected, and analyzed using flow cytometry (excitation at 488 nm, emission at 525 nm). FlowJo software was used for data processing and analysis.

**Cytotoxicity:**

The cytotoxicity of NPs across different cell lines was assessed using the methyl thiazolyl tetrazolium (MTT) assay. Briefly, cells were seeded in 96-well plates and cultured for 24 hours. Subsequently, the cells were exposed to NPs at gradient concentrations (0, 5, 10, 25, 50, 100, 200, and 400 μg/mL) and incubated for an additional 24 hours. Following treatment, the culture medium was removed and replaced with fresh medium containing MTT (0.5 mg/mL). After a 4-hour incubation, 150 μL of dimethyl sulfoxide (DMSO) was added to dissolve the formazan crystals, and the absorbance at 492 nm was measured using a microplate reader.

**Mitochondrial membrane potential:**

The mitochondrial membrane potential was evaluated using the JC-1 fluorescent probe (5,5',6,6'-tetrachloro-1,1',3,3'-tetraethylbenzimidazolylcarbocyanine iodide). Under conditions of high membrane potential, JC-1 accumulates in the mitochondrial matrix and forms red fluorescent aggregates. In contrast, when the membrane potential is low due to mitochondrial damage, JC-1 remains in its monomeric form, emitting green fluorescence. Cells were seeded in 12-well plates for 24 hours, followed by treatment with H_2_O_2_ (600 μM) to induce oxidative stress. Different formulations were then added, and the cells were further incubated for 24 hours. The cells were subsequently stained with JC-1 at 37 ℃ for 30 minutes. After three washes with PBS, fluorescence images were captured using a laser confocal microscope. The ratio of red to green fluorescence intensity was calculated for statistical analysis.

**In vitro BBB transmembrane simulation:**

An in vitro monolayer blood-brain barrier (BBB) model was established using Transwell inserts. Endothelial cells were seeded in the upper chamber and cultured for 7 days. When the transendothelial electrical resistance (TEER) value exceeded 200 Ω·cm^2^, indicating barrier formation, PC12 cells were seeded in the lower chamber. After 24 hours of co-culture, TEER measurements were performed to confirm barrier integrity. Nanomaterials (50 μg/mL) were then added to the upper chamber and incubated for 4 hours. The translocation efficiency was assessed by observing the fluorescence intensity of Rhodamine B (RhB) in PC12 cells using an inverted fluorescence microscope.

**Cell apoptosis:**

The apoptotic effect was evaluated by fluorescence staining and flow cytometry analysis. PC12 cells were seeded in six-well plates at a density of 4 × 10^4^ cells/mL and allowed to adhere. Subsequently, the cells were treated with 0.2 mM H_2_O_2_ and the test samples, and incubated at 37 ℃ for 24 hours. After treatment, the culture medium was removed, and the cells were rinsed with PBS. For fluorescence imaging, Calcein-AM dye was added and incubated at 37 ℃ in the dark for 30 minutes, followed by PI staining at room temperature in the dark for 5 minutes. Cell morphology was observed using a fluorescence microscope.

**Flow cytometry analysis:**

Following treatment, cells were collected by resuspending in binding buffer and stained with Annexin V-FITC and/or PI according to the manufacturer's instructions. In the single staining control groups, 5 μL of either Annexin V or PI was added separately; in the double staining group, both dyes were added simultaneously. The stained cells were analyzed using a flow cytometer, and the apoptosis rate and type were determined based on the fluorescence signals of Annexin V (green) and PI (red).

**In vitro iNOS inhibition assay (Griess assay):**

To assess the iNOS inhibitory potential of the materials, RAW264.7 macrophages were seeded in 96-well plates at a density of 1 × 10^5^ cells/well and stimulated with lipopolysaccharide (LPS, 1 μg/mL) and recombinant mouse interferon-γ (IFN-γ, 100 U/mL). The test samples were co-incubated with the cells for 24 hours. Subsequently, the supernatants were collected and analyzed using the Greiss reagent to quantify nitrite levels, a marker of NO production. After adding the Greiss reagent, the mixture was incubated in the dark for 10 minutes, and the absorbance at 540 nm was measured using a microplate reader. The iNOS inhibition percentage was calculated by subtracting the baseline absorbance of the medium and normalizing to the maximum nitrite signal from the stimulated control group.

**Animals:**

Male Sprague-Dawley (SD) rats weighing 220–250 g were used in this study. The animals were housed under controlled conditions with a 12-hour light/dark cycle, ambient temperature of 20–26 ℃, and humidity of 40–70%. Food and water were provided ad libitum. All experimental procedures were conducted in accordance with the NIH Guide for the Care and Use of Laboratory Animals and approved by the Animal Ethics and Experimental Committee of Jilin University (Approval No. 2024 extension No. 552). The middle cerebral artery occlusion (MCAO) model was induced using a silicone-coated nylon suture (Beijing Xiongcong Biotechnology Co., Ltd.). Rats were anesthetized using a small animal gas anesthesia machine and maintained with 2% isoflurane. The right common carotid artery, internal carotid artery and external carotid artery were exposed by dissection. After ligation of the distal end of the external carotid artery, a suture was inserted into the bifurcation of the right common carotid artery to the origin of the right middle cerebral artery (insertion depth about 18-20 mm) to block blood flow. After 2 hours of ischemia, the filament was withdrawn to allow reperfusion. The sham-operated group underwent the same surgical procedure without arterial occlusion. The MCAO model rats were randomly divided into four groups: sham group, AHA group, PBB group, and PBB@AHA group.

**Small animal in vivo imaging:** ICG-labeled nanoparticles (20 mg/kg) were intravenously administered to MCAO rats, and their biodistribution was monitored at various time points using a small animal fluorescence imaging system (Newton 7.0, Vilver, France).

**TTC staining:** 2,3,5-Triphenyltetrazolium chloride (TTC) is enzymatically reduced to a red formazan product by dehydrogenases in viable cells. In contrast, infarcted areas remain unstained due to loss of enzymatic activity. Twenty-four hours after MCAO, rat brains were harvested and sectioned into 2 mm-thick coronal slices from the frontal pole. The slices were incubated in 2% TTC solution at 37 ℃ for 30 minutes, fixed, and photographed using a digital camera. The infarct volume was quantified using ImageJ software.

**Determination of inflammatory factors in brain tissue:** The levels of pro- and anti-inflammatory cytokines, including IL-10, TGF-β, IL-6, and TNF-α, in brain tissue were quantified using ELISA kits. Twenty-four hours post-stroke, MCAO rats were deeply anesthetized and transcardially perfused with saline to remove blood. Brain tissues were collected, homogenized, and centrifuged at 12,000 rpm for 10 minutes to obtain the supernatant. Protein concentration was determined using a BCA protein assay kit. Cytokine levels were then measured according to the manufacturer’s instructions.

**Neurobehavioral tests:** Neurological deficits were assessed 24 hours after MCAO using the internationally recognized Loga neurological scoring system. An independent investigator blinded to the experimental groups performed the evaluation. Rats that died during the experiment were excluded from the analysis. Scoring criteria were as follows: 0 – no deficit; 1 – mild deficit (incomplete extension of the contralateral forelimb); 2 – moderate deficit (circling toward the contralateral side); 3 – severe deficit (leaning to the contralateral side); 4 – very severe deficit (no spontaneous walking and loss of consciousness).

**Immunohistochemical analysis:** Following transcardial perfusion with PBS and 4% paraformaldehyde, brain tissues were collected and processed for both frozen and paraffin sectioning. Paraffin sections were stained with hematoxylin and eosin (H&E) to evaluate histopathological changes. Terminal deoxynucleotidyl transferase dUTP nick end labeling (TUNEL) assay was performed to detect apoptotic cells. The number and morphology of Nissl bodies, which reflect neuronal integrity and recovery in the hippocampus, were assessed using Nissl staining. Additionally, immunofluorescence staining was carried out using neuronal nuclei (NeuN) antibody to label mature neurons. To evaluate microglial polarization, the aforementioned CD86 (M1 marker, Solarbio, Catalog No.: K010082P, 1:500) and CD206 (M2 marker, Solarbio, Catalog No.: K011692M, 1:4000) antibodies were used, followed by AlexaFluor647- and FITC-conjugated secondary antibodies, respectively. Paraffin sections were also stained with Iba-1 antibody (Proteintech, Catalog No.: 81728-1-RR, 1:1000) to identify microglia and assess anti-inflammatory responses.

**In vivo biological safety evaluation:** Major organs, including the heart, liver, spleen, lungs, and kidneys, were collected from treated rats after euthanasia and subjected to histological examination via hematoxylin and eosin (H&E) staining to assess potential systemic toxicity.


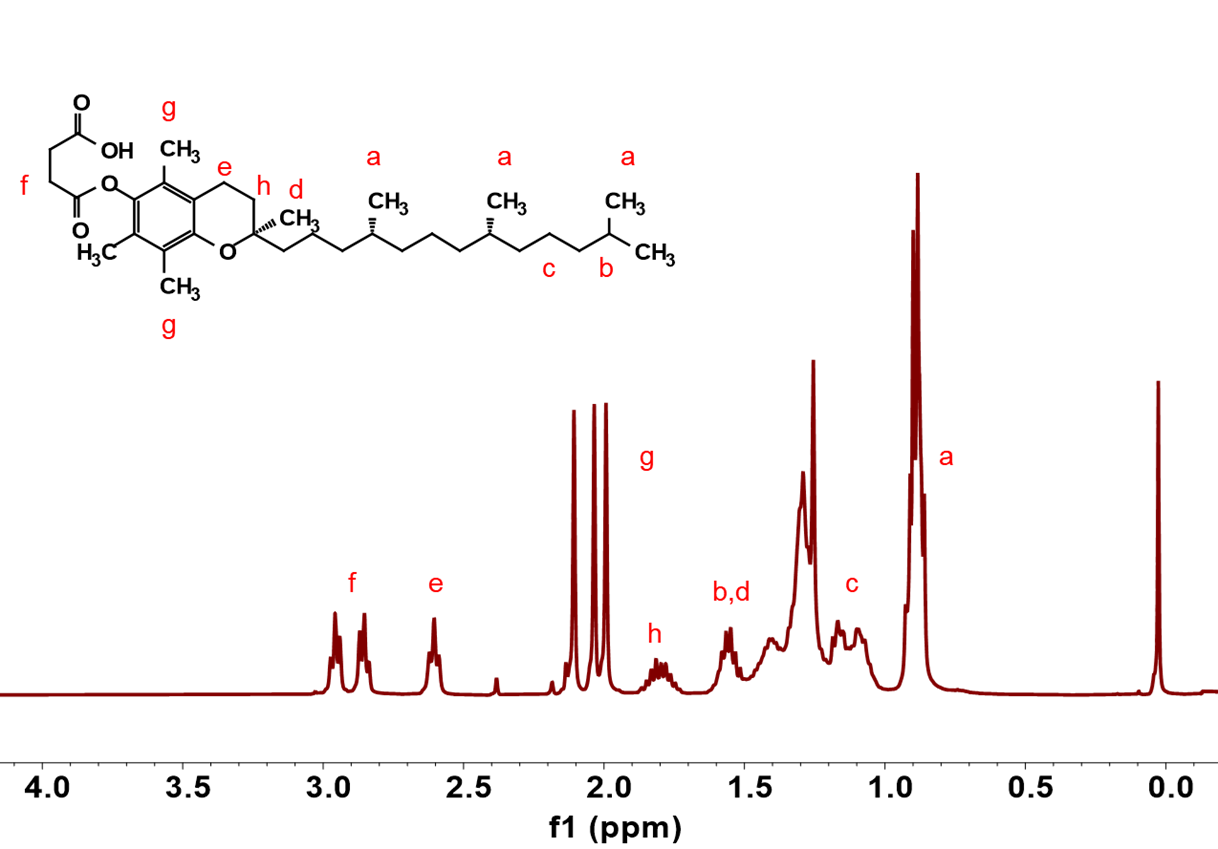


Figure S1. ^1^H NMR spectrum of VES in CDCl_3_.


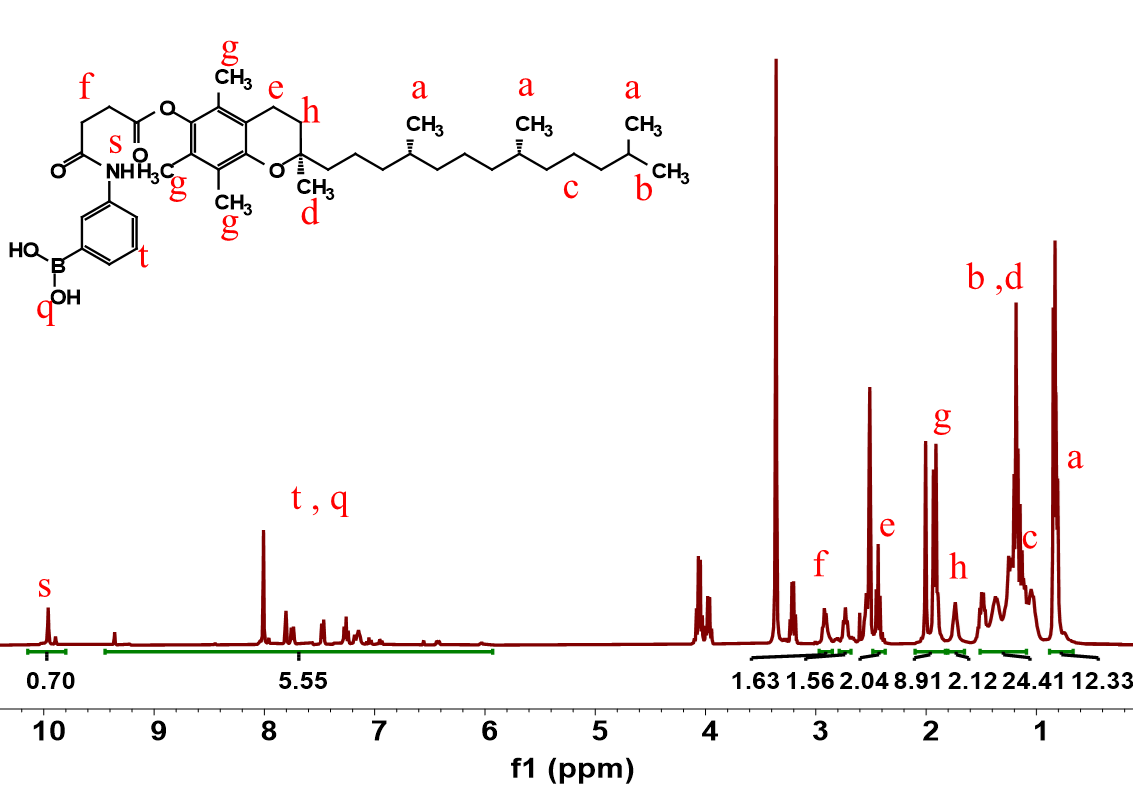


Figure S2. ^1^H NMR spectrum of VES-APBA in CD_3_OD.


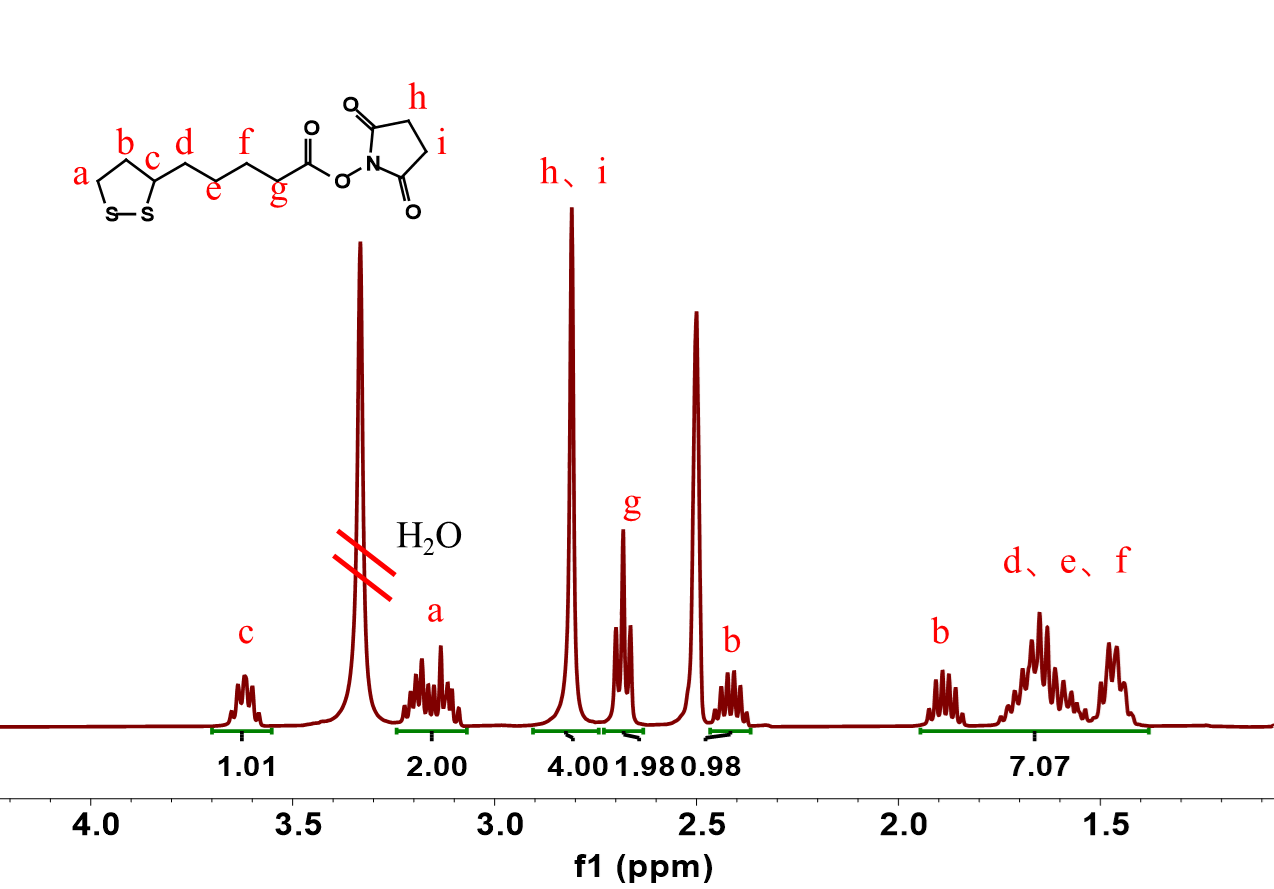


Figure S3. ^1^H NMR spectrum of LA-NHS in (CD_3_)_2_SO.
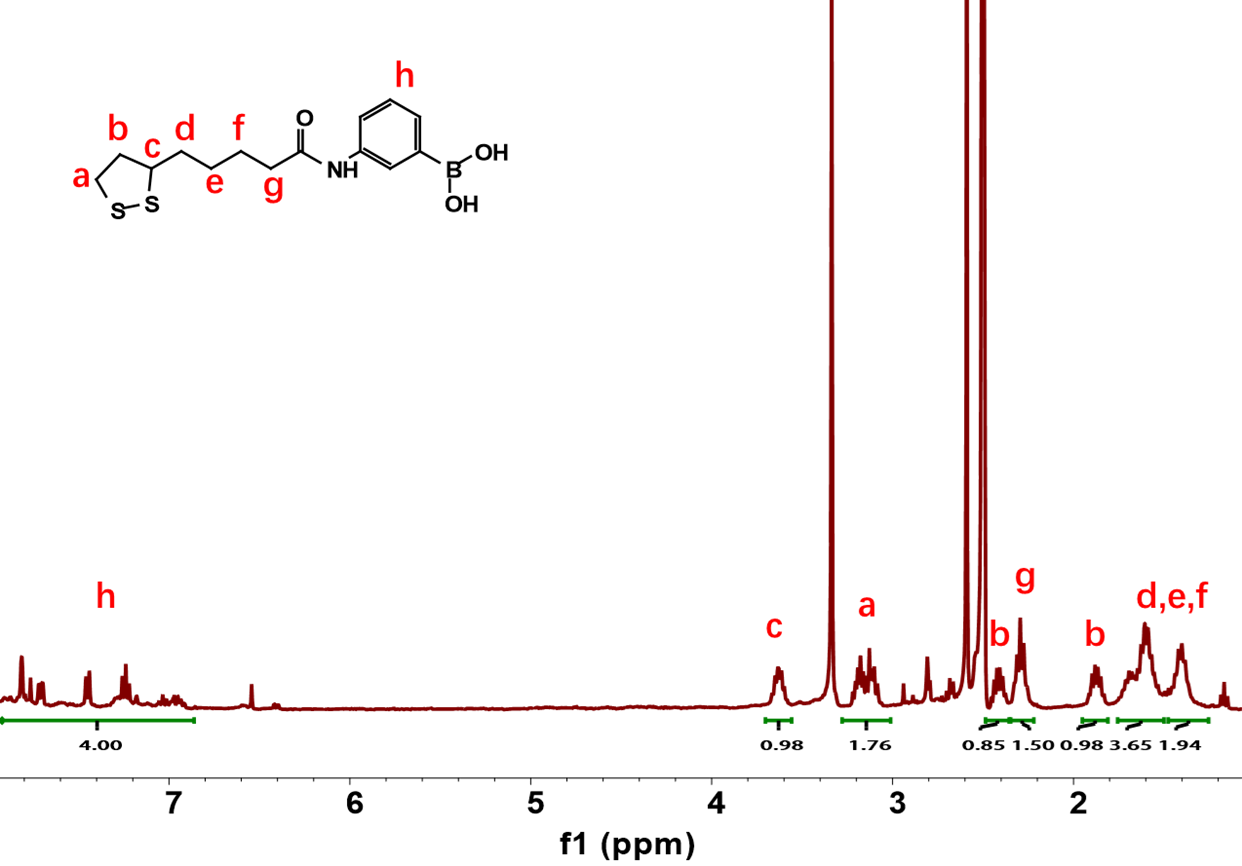


Figure S4. ^1^H NMR spectrum of LA-APBA in CD_3_OD.


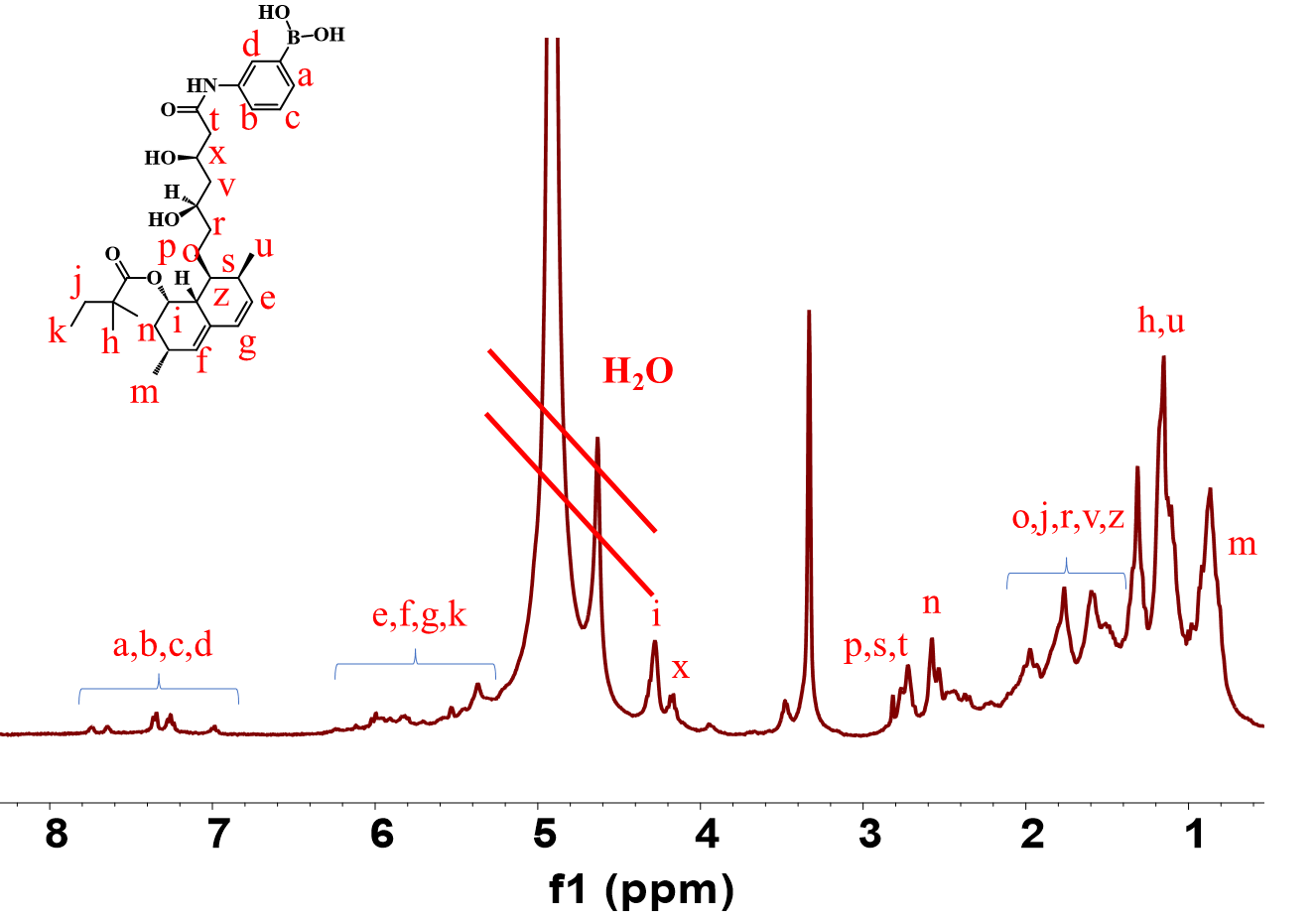
Figure S5. ^1^H NMR spectrum of SA-APBA in CD_3_OD.
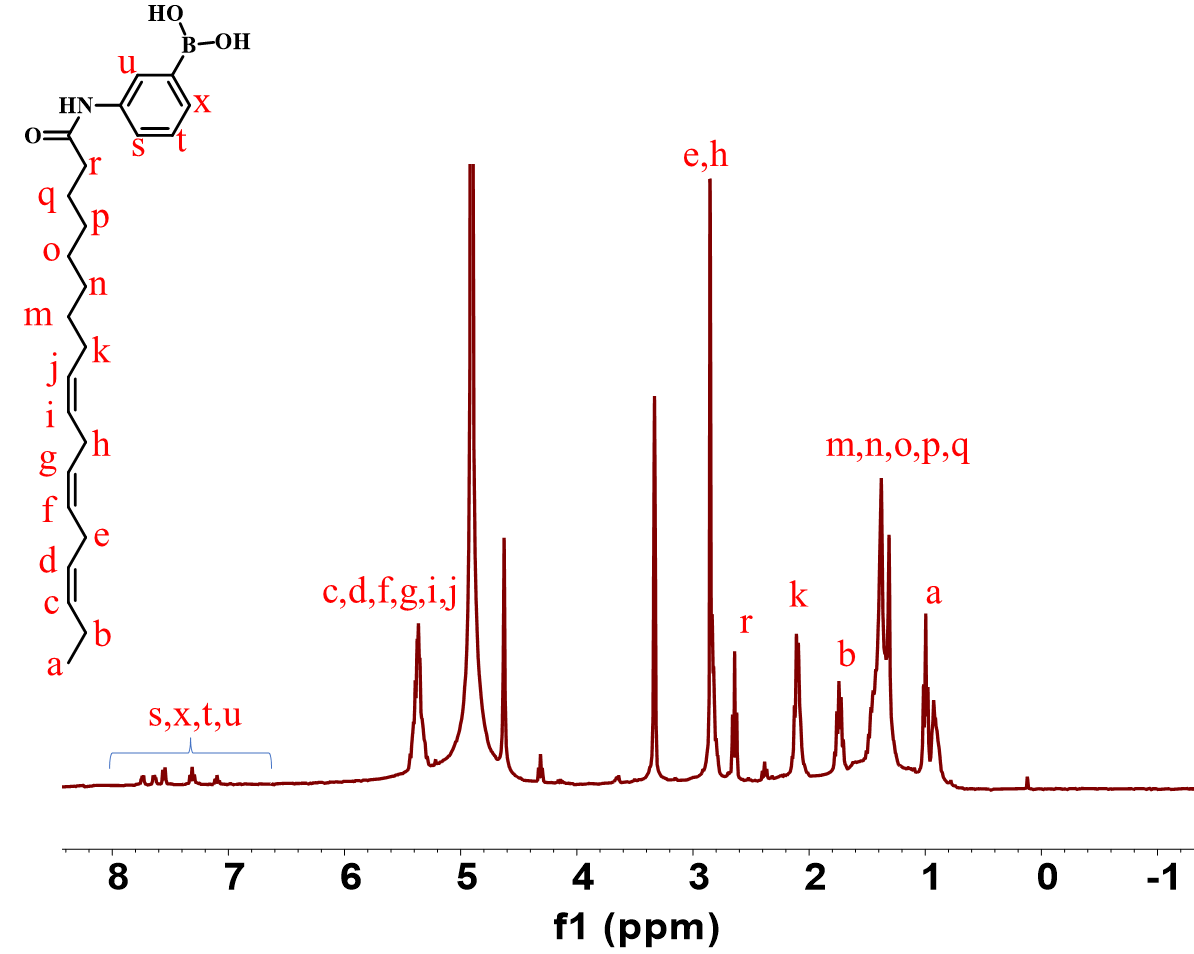


Figure S6. ^1^H NMR spectrum of Omega-3-APBA in CD_3_OD.


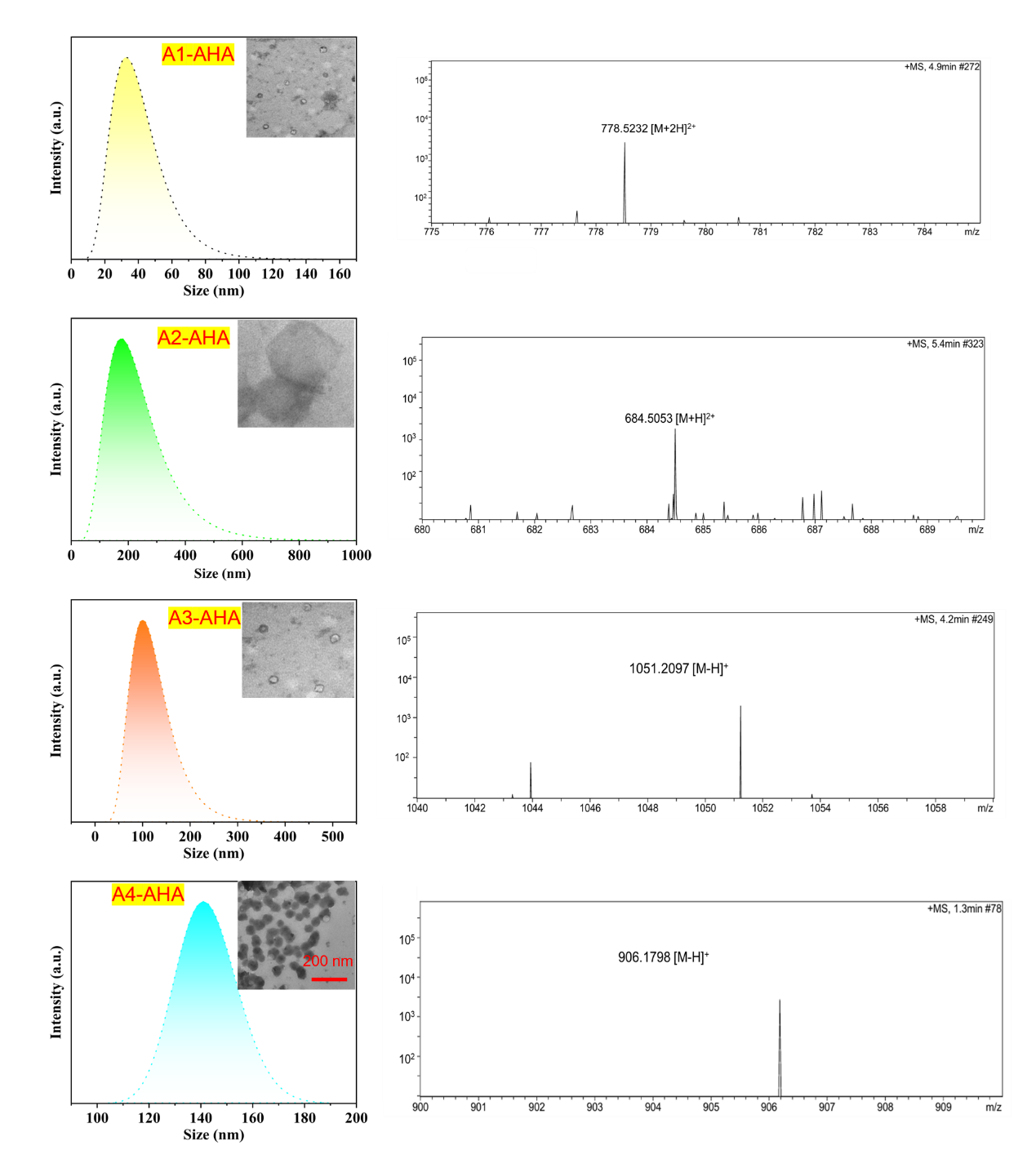


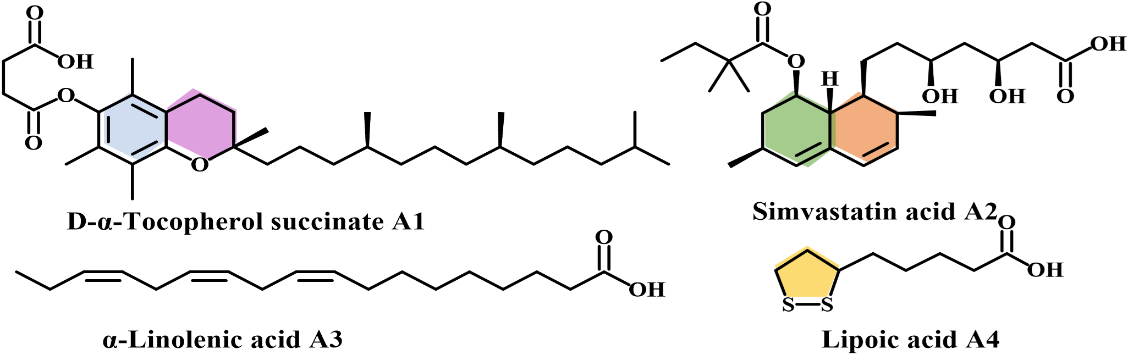


Figure S7. shows the AHA formed by self-assembly of A1-A4 after chemical synthesis. The particle size distribution was determined by Malvern particle size analyzer and TEM, and the corresponding mass spectrum was obtained by high performance liquid chromatography-mass spectrometry (scale bar = 200 nm).


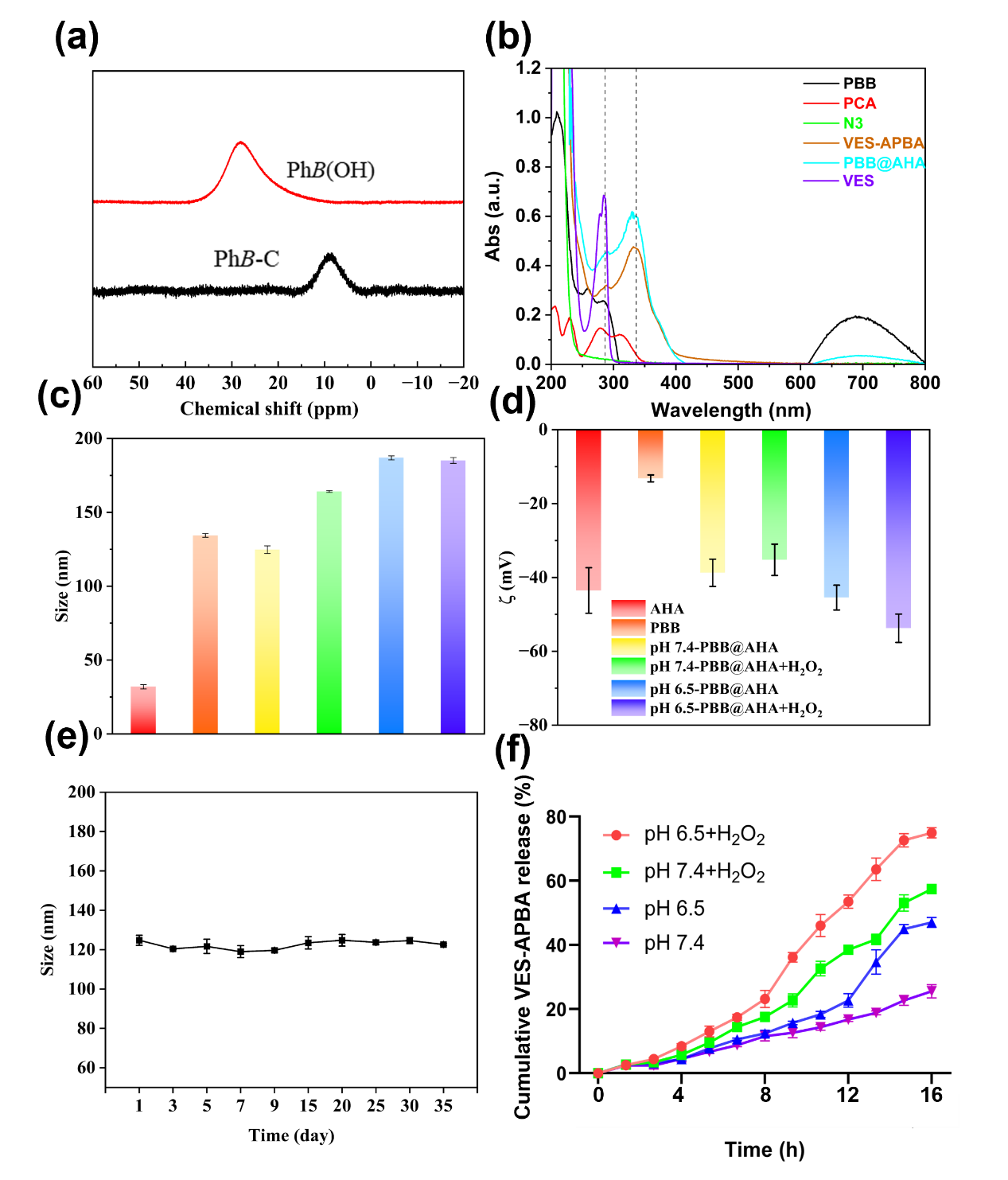


Figure S8. Characterization of VES-APBA and PBB@AHA formulations. (a) ^11^B NMR spectra of VES-APBA and PBB@AHA. (b) UV–vis absorption spectra of PBB, PCA, N3, VES-APBA, PBB@AHA, and VES in PBS (10 mM, pH 7.4). (c) Particle size and (d) zeta potential of AHA, PBB, and PBB@AHA in various PB buffer solutions (n = 3). (e) Particle size stability of PBB@AHA over 35 days (n = 3). (f) In vitro drug release profile of PBB@AHA (n = 3).


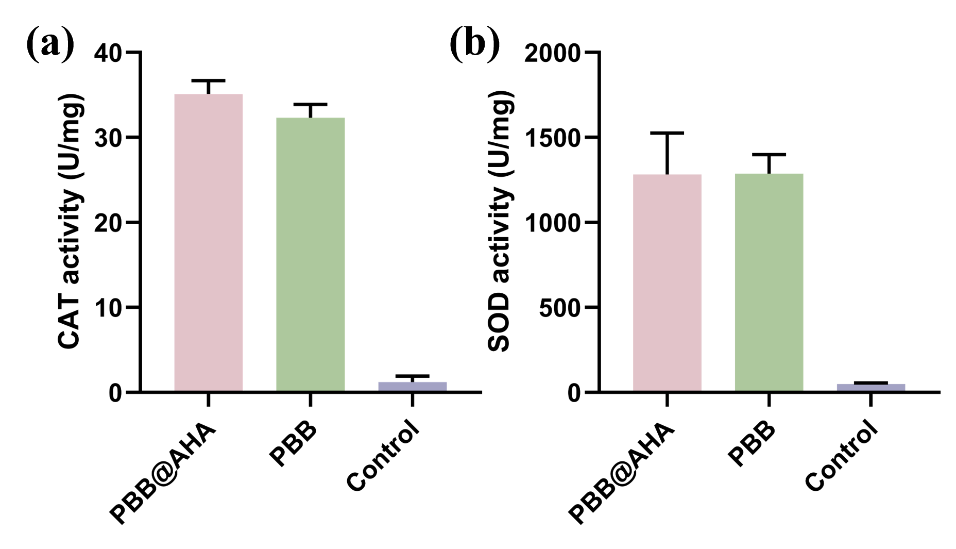


Figure S9. (a) CAT and (b) SOD activity of PBB@AHA .


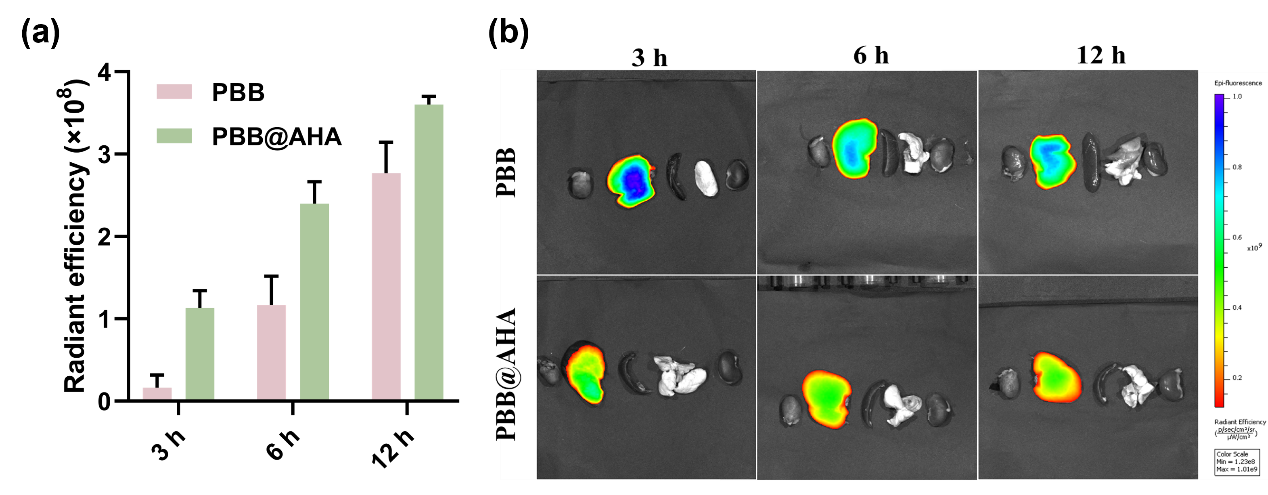


Figure S10. Quantitative fluorescence assay results of rat brains(a) and in vivo imaging(b) of major organs at 3, 6, and 12 hours post-treatment across different therapeutic groups.


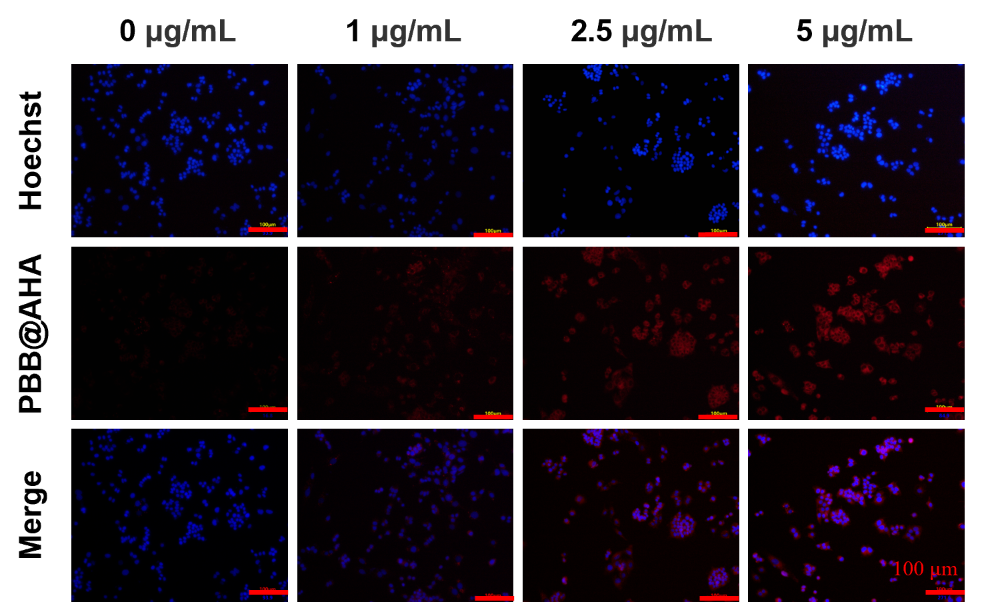


Figure S11. Cellular uptake of Nile Red-labeled PBB@AHA by PC12 cells at varying concentrations (scale bar = 100 μm).


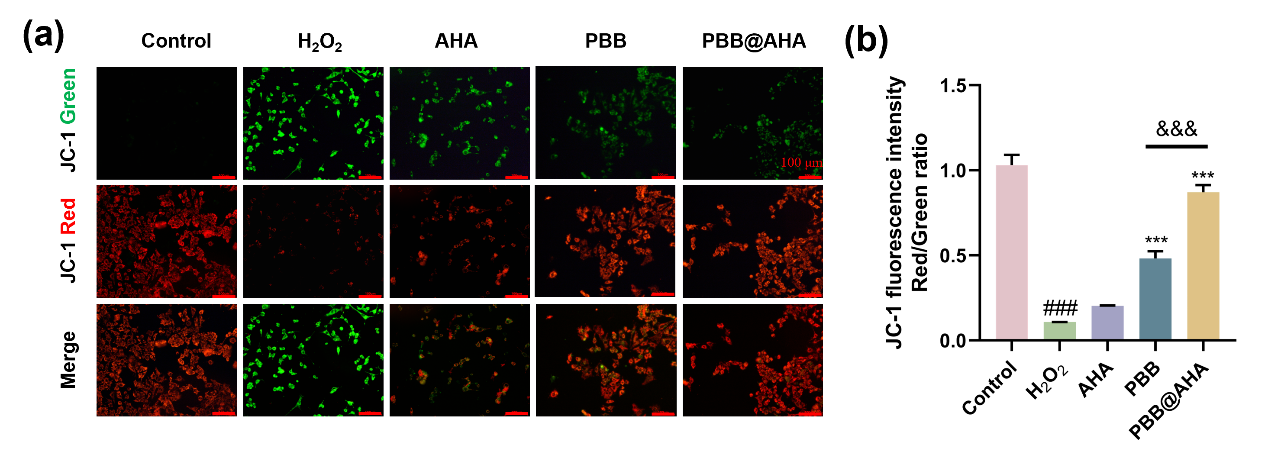


Figure S12. (a) Representative images of JC-1 fluorescence staining obtained using inverted fluorescence microscopy (scale bar = 100 μm). (b) Ratio of JC-1 aggregates (red) to JC-1 monomers (green) fluorescence, analyzed based on inverted fluorescence microscopy images, used to evaluate mitochondrial membrane potential (n = 4). Significant differences between the control group and the H_2_O_2_/induced group are indicated as ^#^P < 0.05, ^##^P < 0.01, ^###^P < 0.001; ^*^P < 0.05, ^**^P < 0.01, ^***^P < 0.001 compared with the H_2_O_2_/induced group; and ^&^P < 0.05, ^&&^P < 0.01, ^&&&^P < 0.001.


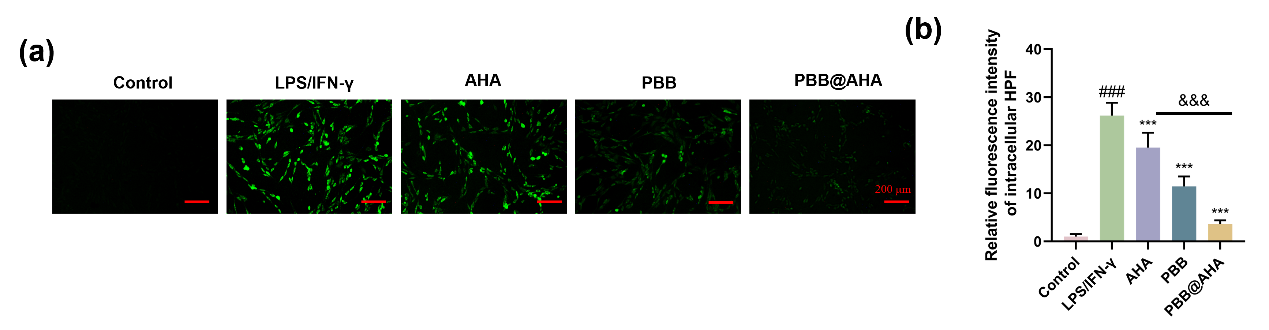


Figure S13. Representative images of intracellular reactive oxygen species levels detected using the HPF fluorescent probe (a, scale bar = 200 μm) and corresponding fluorescence quantification data (b).


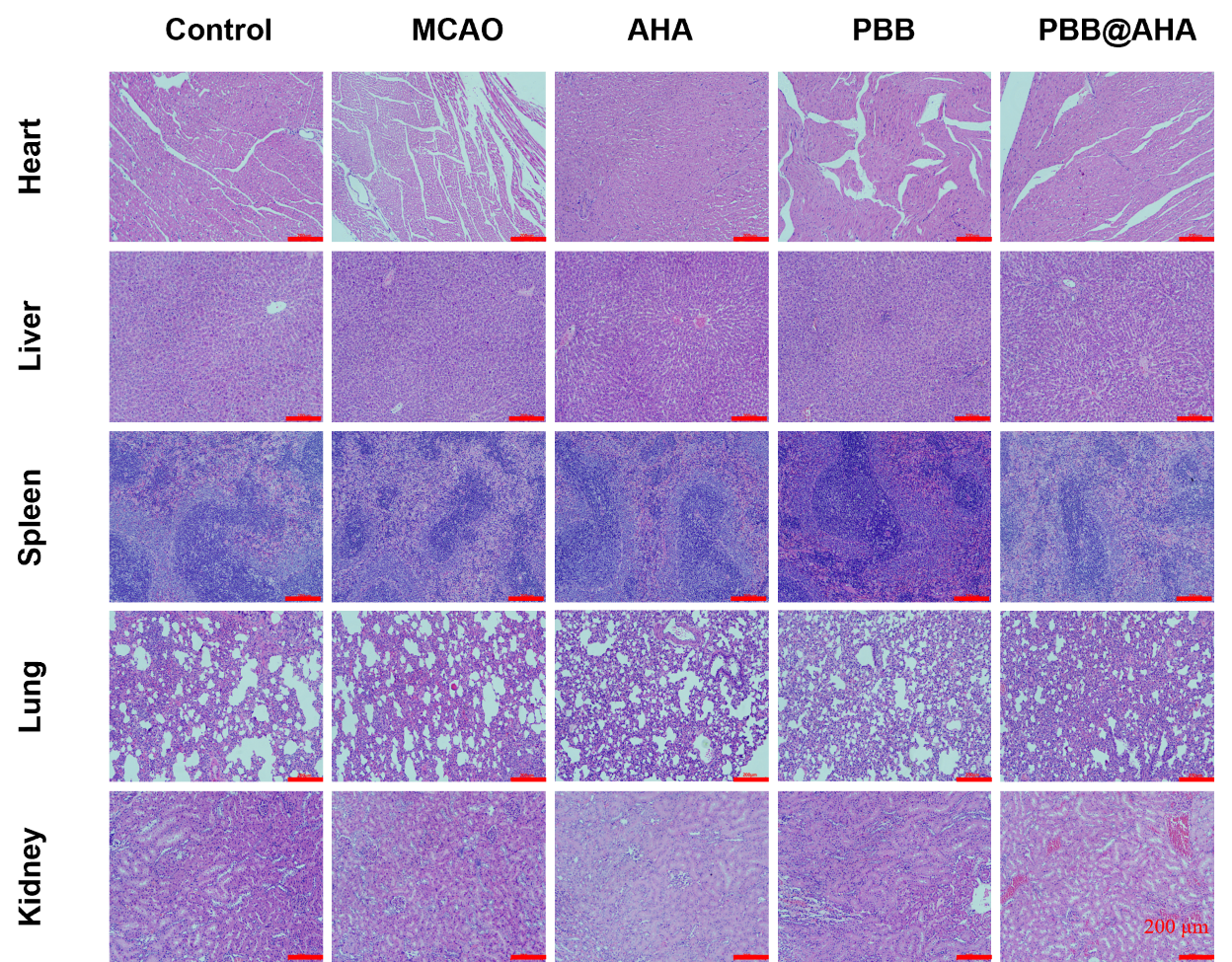


Figure S14. H&E staining results of heart, liver, spleen, lung and kidney tissues after treatment in different treatment groups (scale bar = 200 μm).


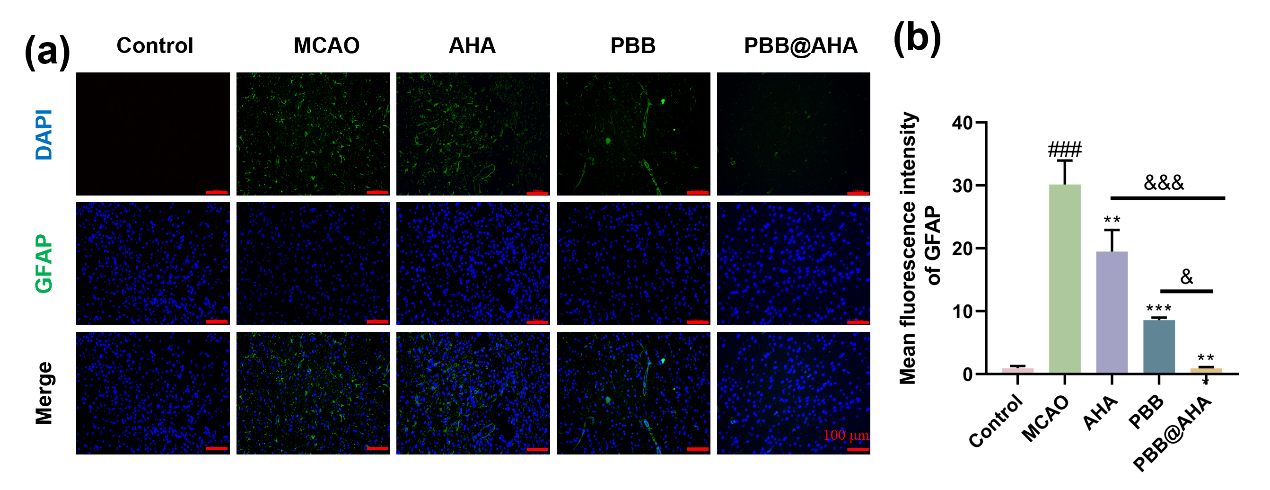


Figure S15. GFAP-stained tissue slice images (a) and corresponding immunofluorescence intensity analysis (b) from different treatment groups (scale bar = 100 μm).


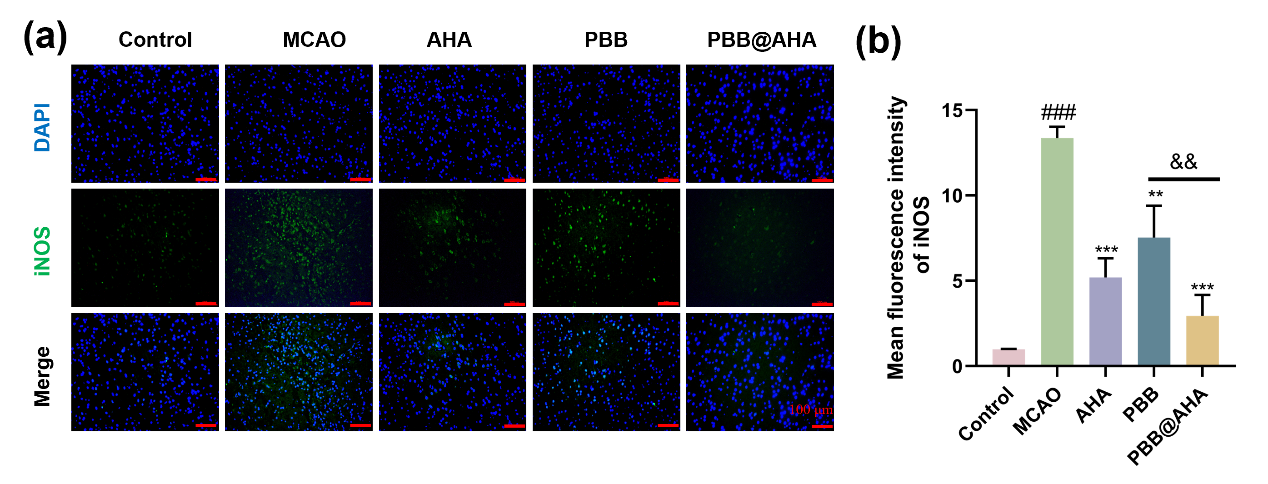


Figure S16. Immunofluorescence images (a, scale bar = 100 μm) and quantified iNOS immunofluorescence intensity (b) across groups 24 hours post-stroke. Significant differences between the control group and the MCAO/Model group are indicated as ^#^P < 0.05, ^##^P < 0.01, ^###^P < 0.001; ^*^P < 0.05, ^**^P < 0.01, ^***^P < 0.001 compared with the MCAO /induced group; and ^&^P < 0.05, ^&&^P < 0.01, ^&&&^P < 0.001.
